# A Translocated Effector Required for *Bartonella* Dissemination from Derma to Blood Safeguards Migratory Host Cells from Damage by Co-translocated Effectors

**DOI:** 10.1371/journal.ppat.1004187

**Published:** 2014-06-19

**Authors:** Rusudan Okujava, Patrick Guye, Yun-Yueh Lu, Claudia Mistl, Florine Polus, Muriel Vayssier-Taussat, Cornelia Halin, Antonius G. Rolink, Christoph Dehio

**Affiliations:** 1 Focal Area Infection Biology, Biozentrum, University of Basel, Basel, Switzerland; 2 Unité Sous Contrat *Bartonella*, Institut national de la recherche agronomique (INRA), Maisons-Alfort, France; 3 Institute of Pharmaceutical Sciences, ETH, Zurich, Switzerland; 4 Department of Biomedicine (DBM), University of Basel, Basel, Switzerland; University of California, Davis, United States of America

## Abstract

Numerous bacterial pathogens secrete multiple effectors to modulate host cellular functions. These effectors may interfere with each other to efficiently control the infection process. *Bartonellae* are Gram-negative, facultative intracellular bacteria using a VirB type IV secretion system to translocate a cocktail of *Bartonella* effector proteins (Beps) into host cells. Based on *in vitro* infection models we demonstrate here that BepE protects infected migratory cells from injurious effects triggered by BepC and is required for *in vivo* dissemination of bacteria from the dermal site of inoculation to blood. Human endothelial cells (HUVECs) infected with a Δ*bepE* mutant of *B. henselae* (*Bhe*) displayed a cell fragmentation phenotype resulting from Bep-dependent disturbance of rear edge detachment during migration. A Δ*bepCE* mutant did not show cell fragmentation, indicating that BepC is critical for triggering this deleterious phenotype. Complementation of Δ*bepE* with BepE*_Bhe_* or its homologues from other *Bartonella* species abolished cell fragmentation. This cyto-protective activity is confined to the C-terminal *Bartonella* intracellular delivery (BID) domain of BepE*_Bhe_* (BID2.E*_Bhe_*). Ectopic expression of BID2.E*_Bhe_* impeded the disruption of actin stress fibers by Rho Inhibitor 1, indicating that BepE restores normal cell migration via the RhoA signaling pathway, a major regulator of rear edge retraction. An *intradermal* (*i.d.*) model for *B. tribocorum (Btr)* infection in the rat reservoir host mimicking the natural route of infection by blood sucking arthropods allowed demonstrating a vital role for BepE in bacterial dissemination from derma to blood. While the *Btr* mutant Δ*bepDE* was abacteremic following *i.d.* inoculation, complementation with BepE*_Btr_*, BepE*_Bhe_* or BIDs.E*_Bhe_* restored bacteremia. Given that we observed a similar protective effect of BepE*_Bhe_* on infected bone marrow-derived dendritic cells migrating through a monolayer of lymphatic endothelial cells we propose that infected dermal dendritic cells may be involved in disseminating *Bartonella* towards the blood stream in a BepE-dependent manner.

## Author Summary

Cell migration, a fundamental feature of eukaryotic cells, plays a crucial role in mounting an effective immune response. However, several pathogens subvert the migratory properties of infected host cells to their benefit, such as using them as Trojan horses to disseminate within the host. *Bartonella* effector proteins (Beps) are *bona fide* virulence factors indispensable for the colonization of mammalian target cells. However, their multiple interferences with host cellular signaling processes might culminate in deleterious secondary effects that require additional effectors to maintain the host cell integrity. A striking example is BepE, which is shown here to preserve endothelial cells (ECs) from fragmentation and to inhibit the defects of dendritic cell (DCs) migration caused by BepC and possibly other Beps. Moreover, BepE is essential for *Bartonella* dissemination from the dermal site of inoculation to the blood stream where bacteria establish long-lasting intraerythrocytic bacteremia as a hallmark of infection in the mammalian reservoir host. Migration of *Bartonella*-infected DCs through a monolayer of lymphatic ECs was also found to be dependent of BepE, suggesting that BepE is required to preserve the migratory capability of DCs, a candidate cell type for systemic dissemination from the dermal site of inoculation.

## Introduction

Pathogenic bacteria have evolved a multitude of virulence factors in order to manipulate the host to evade immune responses and to reach their replicative niche - a safe compartment to proliferate that is also a prerequisite for transmissibility [1]. Translocation of bacterial effector proteins into host cells is one of the mechanisms to manipulate the host by interfering with its signaling pathways. A prominent example is CagA, a multifunctional effector protein of the *Helicobacter pylori* (*Hpy*) type IV secretion system (T4SS). CagA modulates both innate and adaptive immune responses of the host and assists *Hpy* to infect the gastric mucosa in about half of the world population for their lifetime [2], [3]. Numerous effector proteins of *Salmonella* type III secretion systems (T3SS) SPI1 and SPI2 [1] and *Shigella* T3SS play a critical role in invasion of non-phagocytic intestinal cells, for further dissemination and modulation of the host inflammatory responses [4], [5]. In addition to targeting the host cellular components, some bacteria have evolved effectors that regulate an activity of each other at a specific stage of the host invasion; like *Legionella* Dot/Icm “metaeffector” LubX mediates the degradation of SidH. Or this interplay may happen in an indirect fashion as for many cases of T4SS/T3SS effectors [6].

*Bartonella* species are fastidious, Gram-negative, facultative intracellular bacteria that are highly adapted to a distinct mammalian reservoir host [7], [8], [9], [10]. Infections in the reservoir host range from asymptomatic or sub-clinical (for most animal-specific species) to clinical manifestations with low morbidity and limited mortality, such as human-specific *B. quintana (Bqu)* infections, or even to life-threatening disease, such as human infection by *B. bacilliformis (Bba)* [9], [11]. *Bartonellae* transmission is mediated by blood-sucking arthropod vectors. The strategy involves replication of bacteria in the gut of the arthropod vector and excretion in the feces, with subsequent survival in the environment for several days [12]. The arthropods usually defecate when feeding on mammals and provide a source of local irritation that results in itching, followed by scratching and inoculation of *Bartonella*-containing feces into the derma of the skin [13]. Later, *Bartonella* is known to appear in the blood of the reservoir host, invades erythrocytes as immune-privileged niche and develops long-lasting persistent infections for more than a year for some species [11], [14].

*Bartonellae* evolved two T4SSs (Trw and VirB) while adapting to a wide range of mammalian hosts [15]. Both of them are essential for the interaction with the host but at different stages of the infection cycle [14], [15], [16], [17], [18]. The Trw system seems to mediate host-specific adhesion of *Bartonella* to erythrocytes by binding to the cell surface with its manifold variants of pilus subunits [19], [20], while the VirB system translocates a cocktail of evolutionarily related *Bartonella* effector proteins (Beps) into nucleated host cells. The Beps (named BepA-G in *Bhe*) evolved by duplication of an ancestral *bep* gene followed by their functional diversification and conservation of certain domains and motifs [15]. All Beps have at least one *Bartonella* intracellular delivery (BID) domain and a positively-charged tail in the C-terminus as a signal for translocation through the T4SS [21].

BepE is one of the effector proteins that is conserved within lineage 4, the largest clade within the *Bartonella* genus comprising 11 species, e.g. *B. henselae, B. quintana, B. tribocorum* and *B. grahamii* [15]. *Bhe* BepE (BepE*_Bhe_*) consists of 464 amino acids (aa). The N-terminus of BepE*_Bhe_*, similarly to BepD*_Bhe_* and BepF*_Bhe_*, contains short repeated peptide sequences (EPLYA) with conserved putative tyrosine phosphorylation sites, similar to the EPIYA motif of *Hpy* effector CagA. BepE*_Bhe_* has two BID domains in the C-terminal part [21]. The domains and the motifs but not the spacing in between is well preserved in BepE homologues [15]. Mass spectrometric analysis of BepE*_Bhe_* pull-downs revealed several SH2 domain-containing eukaryotic signaling proteins that interact either with an individual phosphotyrosine of BepE*_Bhe_* within a Csk-like binding motif or two ITIM/ITSM (immunotyrosine inhibitory motif/immunotyrosine switch motif) tandems [22]. Conservation of these specific motifs of BepE*_Bhe_* and the described interaction partners suggest a molecular mimicry of ITIM-containing receptors by bacterial proteins and a potency to interfere with host signaling pathways.

In this study, we identified BepE as an essential bacterial factor for *Bartonella* reservoir host infection via the *intradermal* route resembling natural infection by arthropod vectors, but not for the *intravenous* route. This specific function was assigned to the C-terminal part of BepE*_Bhe_* including the two BID domains. The same BID domains were interfering with a prominent cell fragmentation phenotype in migrating endothelial cell induced by BepC and possibly other Beps as a secondary effect. Further, we show that *Bartonella* translocates an effector-fusion protein (Bla-BID) into dendritic cells and affects cell migration in the absence of BepE, suggesting that DCs may be involved in the BepE-dependent dissemination of *Bartonella* to the blood.

## Results

### BepE*_Bhe_* is sufficient to abolish a cell fragmentation phenotype induced by the *Bhe* Δ*bepDEF* mutant

A previous study on the interactome of *Bartonella* effector protein BepE*_Bhe_* revealed several SH2 domain-containing signaling proteins that interact with BepE*_Bhe_* upon phosphorylation of specific tyrosines within the motifs [22]. Based on these data, we hypothesized that BepE might be a factor impacting multiple cellular signaling pathways to promote the establishment of a successful *Bartonella* infection. To acquire first insights into the cellular phenotypes and uncover the molecular bases of BepE we used the well-established human umbilical vein endothelial cell (HUVEC) infection model for *Bhe* [23], [24], [25], [26], [27].

**Figure 1:**
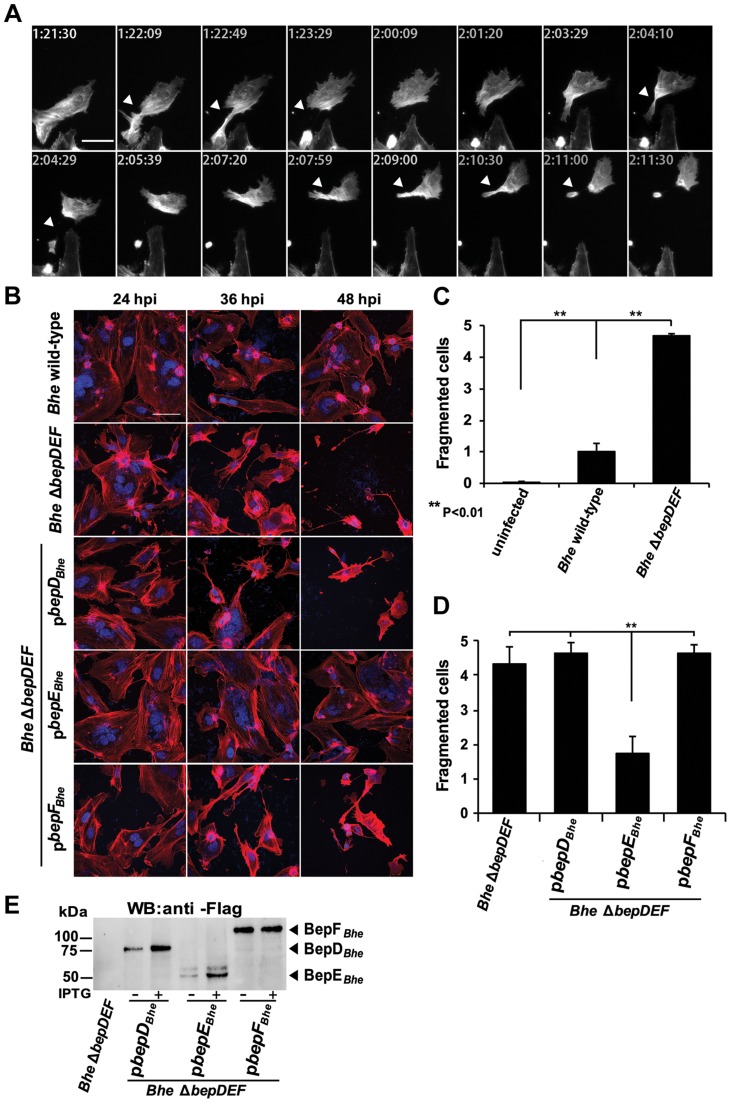
Infection of HUVECs with the *Bhe* Δ*bepDEF* mutant leads to cell fragmentation. Subconfluent monolayers of HUVECs were infected with the indicated bacterial strains at a MOI = 200. (**A**) HUVECs expressing LifeAct-mCherry were infected with *Bhe* Δ*bepDEF* and subjected to live cell imaging with an MD ImageXpress Micro automated microscope. Snapshots of gray scale images taken at different time points as depicted by the time stamps (format: dd: hh:mm) are presented (scale bar = 50 µm). The arrowheads are pointing to the regions of the cell where the fragmentation is taking place. (**B**) HUVECs infected with the indicated bacterial strains were fixed at 24 h, 36 h and 48 h post infection followed by immunocytochemical staining and confocal laser scanning microscopy. F-actin is represented in red and DNA in blue (scale bar = 50 µm). (**C, D**) Quantification of cell fragmentation at 48 hpi was performed in semi-automated manner. Images were acquired in 96 well-plate format by MD ImageXpress Micro automated microscopes with 10× magnification. The number of fragmented cells (cells with thin and multipolar elongations) were defined by eye and counted manually. The percentage of fragmented cells is normalized to *Bhe* wild-type infection. In each condition triplicate wells with each 10 randomly picked fields were imaged and presented as mean +/− SD. Statistical significance was determined using Student's *t*-test. *P*<0.05 was considered statistically significant. Data from one representative experiment (n = 3) are presented. (**E**) Protein levels of the BepD*_Bhe_*, BepE*_Bhe_* and BepF*_Bhe_* by plasmid overexpression in Δ*bepDEF*. The anti-Flag western blot was obtained from total lysate of corresponding *Bhe* strains.

BepE, together with BepD and BepF, belongs to the class of the Beps harboring tyrosine-containing motifs in their N-termini. In order to reduce the complexity of potential redundant functional effects by any of these three tyrosinephosphorylated effectors we infected HUVECs with a *Bhe* mutant carrying an in-frame deletion of the chromosomal region encoding *bepD*, *bepE* and *bepF (Bhe* Δ*bepDEF)*. The infection was studied by means of microscopy of either fixed samples or by time-lapse imaging over 72 h. Starting about 20–24 hours post infection (hpi) the *Bhe* Δ*bepDEF* mutant showed a drastic phenotype of cell fragmentation (Fig. 1A and Movie S1) which was hardly detected in cells infected with wild-type bacteria at this early stage of infection (Fig. 1B, C), but became more apparent at later time points, beyond 48 hpi (Movie S2). Fragmenting cells were apparently normally moving forward but displayed pronounced difficulties in rear end retraction - such cells became more and more elongated and at some point the thin connection between a given cell body and the attached rear broke (Fig. 1A). The fragment left behind migrated on the substrate for a few hours and then came to a halt, while the cell body containing the nucleus got smaller with each fragmentation. Eventually, this process led also to a decreased number of cells within the sample (Fig. 1A and B, Movie S1).

Infections with single effector-complemented *Bhe* Δ*bepDEF* revealed that the cell fragmentation phenotype was inhibited only by expression of BepE*_Bhe_*, while neither BepD*_Bhe_* nor BepF*_Bhe_* displayed a similar cytoprotective effect (Fig. 1B, D).

### A single BID domain of BepE*_Bhe_* is sufficient to interfere with the fragmentation of HUVECs

**Figure 2:**
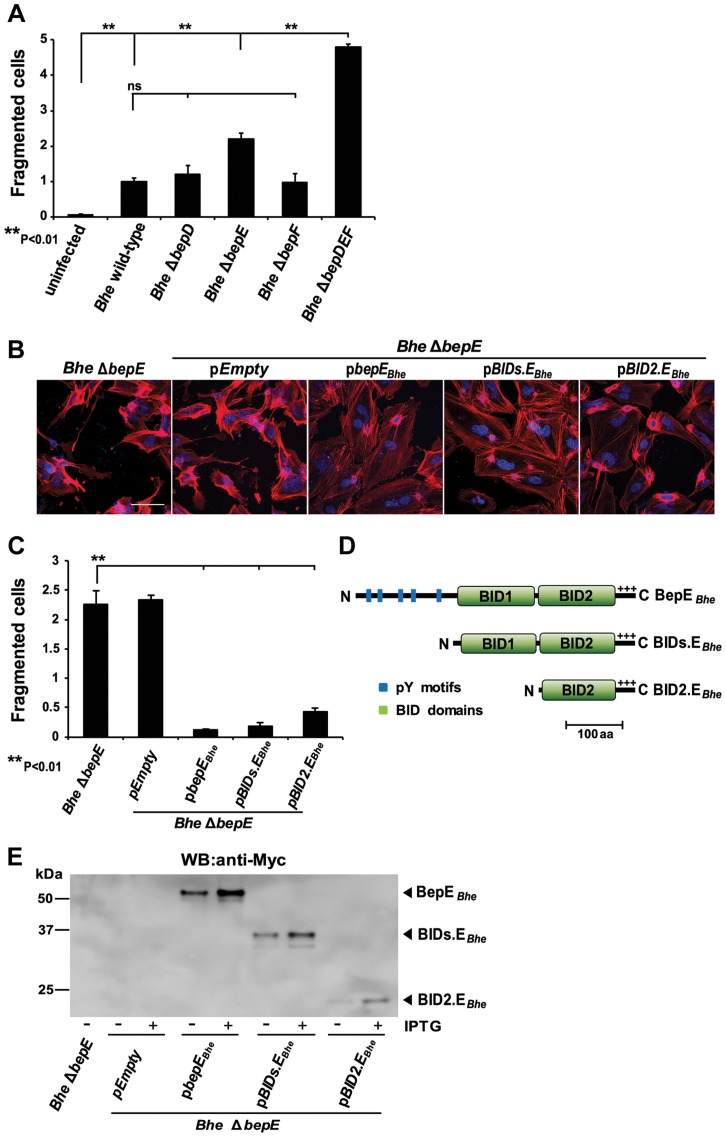
Deletion of BepE is sufficient for *Bhe* to induce cell fragmentation. (**A–C**) Subconfluent monolayers of HUVECs were infected with MOI = 200 of the indicated bacterial strains. (**B**) Infected HUVECs were fixed at 48 h post infection followed by immunocytochemical staining and confocal laser scanning microscopy. F-actin is represented in red and DNA in blue (scale bar = 50 µm). (**A**) and (**C**) quantification of cell fragmentation at 48 h post infection was performed as described for Fig. 1C and D. The mean and SD of triplicate samples is presented. Statistical significance was determined using Student's *t*-test. *P*<0.05 was considered statistically significant. Data from one representative experiment (n = 3) are presented. (**D**) Schematic view of BepE*_Bhe_* and N-terminal deletion mutants expressed in *Bartonella* from a plasmid. (**E**) Protein levels of the BepE*_Bhe_* mutants shown in figure by overexpression in *Bhe* Δ*bepE*. The anti-Myc western blot was obtained from total lysate of corresponding *Bartonella* strains.

Provided that BepD and BepF could not abolish the cell fragmentation phenotype of the Δ*bepDEF* mutant we were interested to test whether single deletions of *bepD, bepE* or *bepF* would be sufficient to trigger cell fragmentation. *Bhe* Δ*bepE*-infected HUVECs showed indeed very similar morphological changes but the effect was less pronounced. Compared to wild-type infection, none of the other mutants could induce elevated fragmentation in a significant manner (Fig. 2A). As expected BepE could also rescue the cell fragmentation led by *Bhe* Δ*bepE* (Fig. 2B and C, Movie S3).

We next delineated the functional domain responsible for the abrogation of cell fragmentation in BepE*_Bhe_*. As indicated earlier, BepE*_Bhe_* has two BID domains at its C-terminus plus a positively charged C-tail. These domains (BID1.E*_Bhe_* and BID2.E*_Bhe_*) show high similarity (pair-wise aa identity of 53.3%) (Fig. S1A) and thus seem to be originated from a duplication event [15]. To understand which part of BepE*_Bhe_* was responsible for the effect observed during the infection of HUVECs, BIDs.E*_Bhe_* and BID2.E*_Bhe_* were expressed in *Bhe* Δ*bepE* as they both contain an intact C-term required for translocation (Fig. 2B–E) and then used to infect HUVECs. The two BID domains (BIDs.E*_Bhe_*) do overcome the *Bhe* Δ*bepE*-induced cell fragmentation phenotype. Even more, BID2.E*_Bhe_* is able to complement with almost the same efficiency as full length BepE.

In summary, these data indicate that the inhibition of cell fragmentation by BepE*_Bhe_* was mediated by the BID domains of BepE*_Bhe_* and more specifically, the BID2.E*_Bhe_* was sufficient.

### Cell fragmentation induced by *Bhe* Δ*bepDEF* is inhibited by heterologous complementation with BepE*_Bhe_* homologues

**Figure 3:**
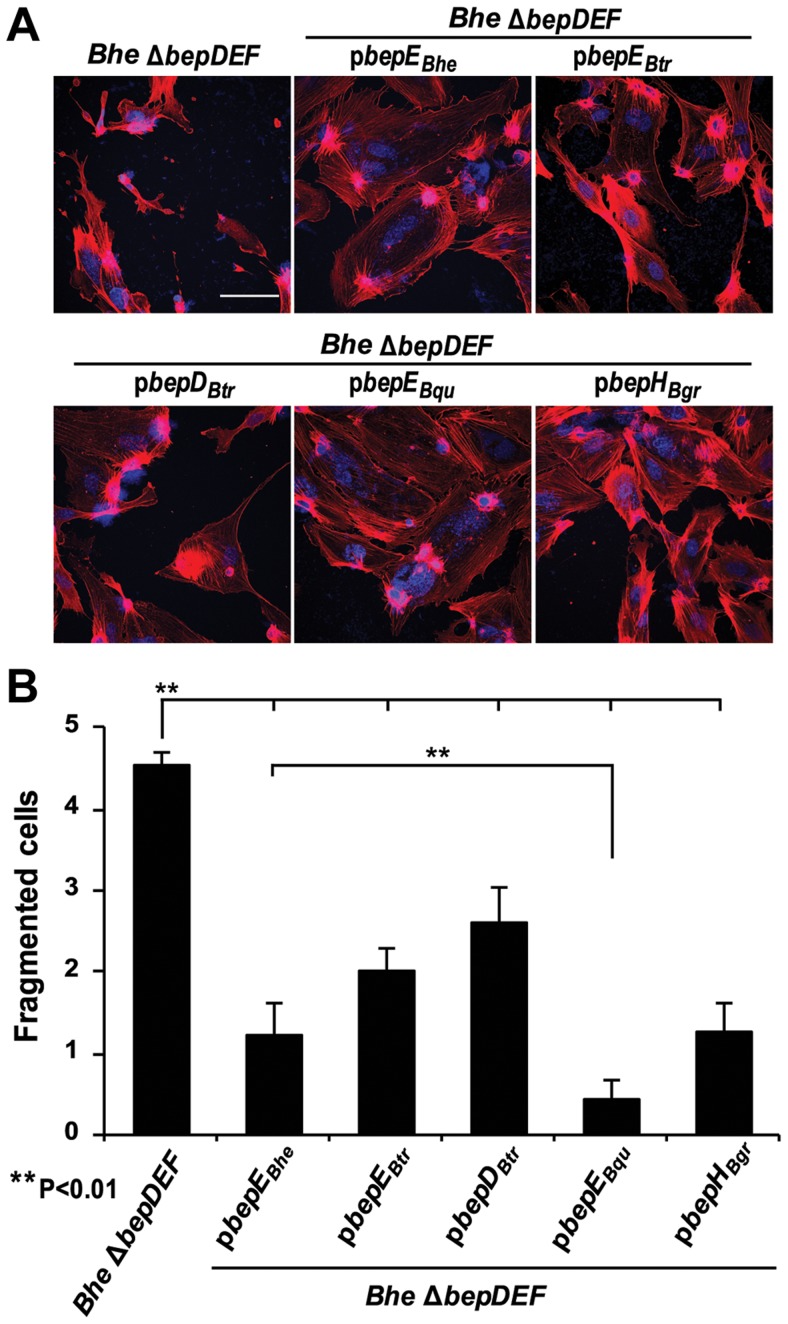
Expression of BepE*_Bhe_* homologues in the *Bhe* Δ*bepDEF* inhibit the cell fragmentation phenotype. (**A**) Subconfluent monolayers of HUVECs were infected for 48 h with MOI = 200 of the *Bhe* Δ*bepDEF* mutant complemented with the indicated Bep-expression plasmids followed by fixation, immunocytochemical staining and confocal laser scanning microscopy. F-actin is represented in red and DNA in blue (scale bar = 50 µm). (**B**) Quantification of cell fragmentation at 48 h post infection was performed as described for Fig. 1C and D and presented as mean of triplicate samples +/− SD. Statistical significance was determined using Student's *t*-test. *P*<0.05 was considered statistically significant. Data from one representative experiment (n = 3) are presented.

BepE homologues from the *Bartonella* lineage 4 species display significant similarity in domain and motif composition (Fig. S1B). We thus tested whether BepE homologues from different *Bartonella* species, i.e. BepD*_Btr_* and BepE*_Btr_* from *B. tribocorum*, BepE*_Bqu_* from *B. quintana* and BepH*_Bgr_* from *B. grahamii*, can interfere with the cell fragmentation phenotype of the *Bhe* Δ*bepDEF*. BepE*_Btr_*, BepE*_Bqu_*, and BepH*_Bgr_* were indeed able to functionally replace BepE*_Bhe_* in *Bhe* Δ*bepDEF* background. Amongst all the homologues, BepD*_Btr_* has the least amino similarity to BepE*_Bhe_* (Fig. S1B) and was also the least potent in suppressing the cell fragmentation phenotype (Fig. 3A and B). Interestingly, BepE*_Bqu_* from the human-specific *Bqu* was the most efficient in abrogating cell fragmentation. Considering that HUVECs are primary human endothelial cells this observation is suggestive for some level of host specificity in the activity of the BepE effector.

### Deletion of BepC in the *Bhe* Δ*bepE* mutant background is sufficient to abolish the cell fragmentation phenotype

**Figure 4:**
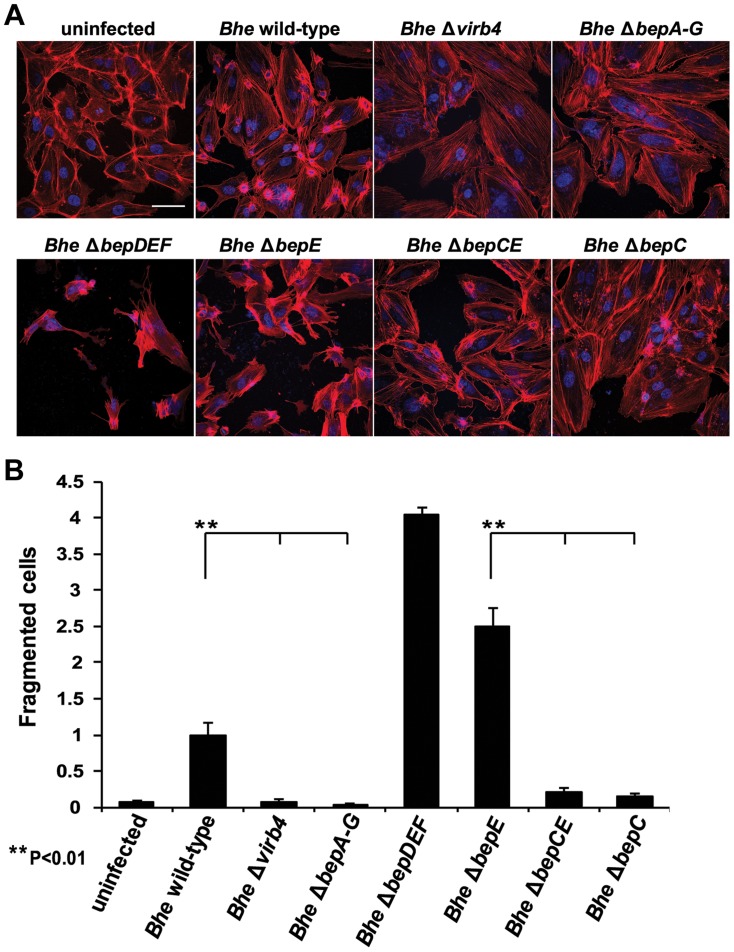
The double deletion mutant *Bhe* Δ*bepCE* abolishes cell fragmentation. (**A**) Subconfluent monolayers of HUVECs were infected for 48 h with MOI = 200 of the *Bhe* strains depicted in the figure or were left uninfected. Samples were then fixed, stained immunocytochemically and analyzed by confocal laser scanning microscopy. F-actin is represented in red (Phalloidin) and DNA in blue (DAPI) (scale bar = 50 µm). (**B**) Quantification of cell fragmentation at 48 h post infection was performed as described for Fig. 1C and D and presented as mean of triplicate samples +/− SD. Statistical significance was determined using Student's *t*-test. *P*<0.05 was considered statistically significant. Data from one representative experiment (n = 3) are presented.

The cell fragmentation phenotype is triggered by the mutant strains *Bhe* Δ*bepE* and *Bhe* Δ*bepDEF*. In order to find the factor from *Bhe* leading to this severe phenotype we tested a set of available *Bhe* mutants. The effector-free mutant *Bhe* Δ*bepA-G* did not display cell fragmentation, indicating that the factor that triggers cell fragmentation in the absence of BepE must be another Bep (Fig. 4A and B). Given that the *Bhe* Δ*bepDEF* mutant strain displays severe cell fragmentation indicates that that the cell fragmentation activity is confined to either BepA, BepB, BepC or BepG or a combination thereof. A double mutant *Bhe* Δ*bepCE* did not display cell fragmentation, indicating that BepC is essential for this activity. Although we cannot exclude that either BepA, BepB, or BepG may contribute to this phenotype, ectopic expression of mCherry-BepC in HUVEC resulted in cell fragmentation that could be reduced by co-expression of GFP-BepE (Movie S4), indicating that BepC can trigger cell fragmentation on its own.

### BepE translocation and interference with cell fragmentation is T4SS-dependent

**Figure 5:**
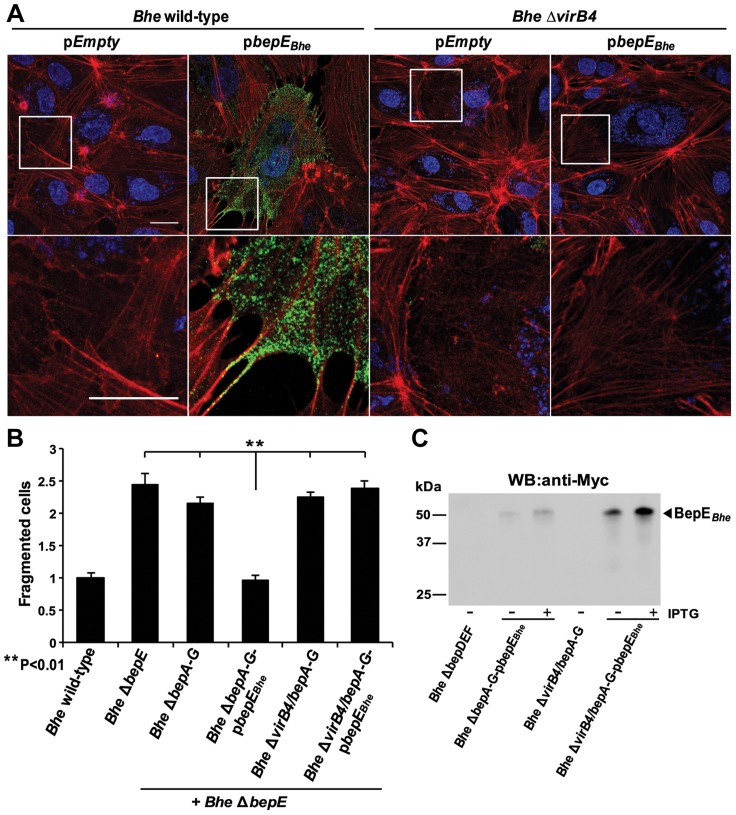
BepE protects host cells from fragmentation upon translocation via T4SS. (**A**) Subconfluent monolayers of HUVECs were infected with MOI = 100 of the indicated bacterial strains for 24 h. After fixation and subsequent immunocytochemical staining the specimen was analyzed by confocal laser scanning microscopy. F-actin is represented in red (Phalloidin) and DNA in Blue (DAPI). Translocation of the effector protein into the infected cells was detected by anti-Myc-staining depicted in green (scale bar = 20 µm). (**B**) Subconfluent monolayers of HUVECs were infected with MOI = 200 or MOI = 200+200 in case of mixed infection depicted in the figure. Quantification of cell fragmentation at 48 h post infection was performed as described for Fig. 1C and D and presented as mean of triplicate samples +/− SD. Statistical significance was determined using Student's *t*-test. *P*<0.05 was considered statistically significant. Data from one representative experiment (n = 2) are presented. (**C**) Protein levels of the BepE*_Bhe_* by overexpression in *Bartonella* strains. The anti-Myc western blot was obtained from total lysate of corresponding *Bartonella* strains depicted in figure.

To confirm that inhibition of cell fragmentation observed upon infection with strains over-expressing BepE from plasmid was fully attributable to BepE effector function within the host cell and not to competitive inhibition with translocation of other Beps, we first confirmed VirB T4SS-dependency of BepE*_Bhe_* translocation into host cells. To this end we expressed Myc-BepE in *Bhe* wild-type or the VirB T4SS-translocation deficient *Bhe* Δ*virB4* mutant strain. Immunocytochemical staining with anti-Myc antibodies showed clearly that BepE*_Bhe_* is translocated in a VirB T4SS-dependent manner (Fig. 5A.) as previously demonstrated for BepD*_Bhe_* [21].

In order to avoid that BepE translocation interferes with BepC and possibly other Beps contributing to the cell fragmentation phenotype, we performed a mixed infection experiment with the strain *Bhe* Δ*bepE* (Fig. 5B) that triggers cell fragmentation and strain expressing BepE in an effector-free mutant background (*Bhe* Δ*bepA-G*-p*BepE*). The results showed that BepE translocated by one strain can suppress the cell fragmentation phenotype mediated by the effectors translocated by the other strain. As a negative control we showed that expression of BepE in a VirB T4SS-deficient background (*Bhe* Δ*virB4*/*bepA-G*-p*bepE*) did not lead to suppression of the cell fragmentation phenotype triggered by the other strain (Fig. 5B, for *Bhe* Δ*bepDEF* see Fig. S2). These data demonstrate that BepE is translocated by the VirB T4SS system into the host cell and upon translocation shows a cytoprotective effect by interfering with the cell fragmentation.

### Ectopic expression of BepE in HUVECs abrogates cell fragmentation

**Figure 6:**
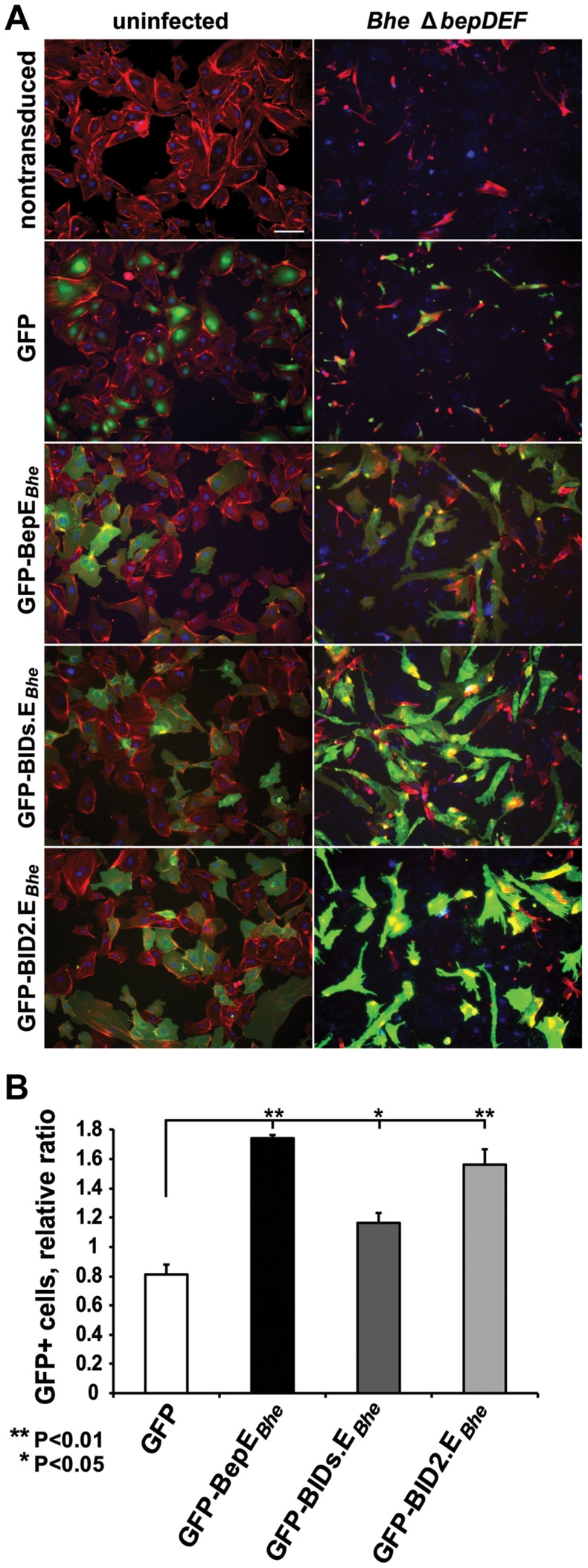
Ectopic expression of BepE*_Bhe_* in HUVECs prevents cell fragmentation. (**A, B**) HUVECs of an early passage were transduced with lentiviruses for the expression of the depicted GFP-fusion proteins. The mixed culture of transduced and non-transduced cells were infected with the indicated *Bhe* strains (MOI = 200). Infected cells were either fixed and stained for microscopy or analyzed for the survival by FACS at 48 hpi. (**A**) Representative microscopy images (scale bar = 100 µm). F-actin is represented in red (Phalloidin), DNA in blue (DAPI), GFP in green. (**B**) Protection by GFP-fused BepE and its derivatives against fragmentation induced by *Bhe* Δ*bepDEF* mutant strains. GFP-positive cell were quantified by FACS and normalized to the uninfected cell population. One representative experiment (n = 3) with the mean of triplicate samples +/− SD are presented. Statistical significance was determined using Student's *t*-test. *P*<0.05 was considered statistically significant.

Next we expressed BepE ectopically in HUVECs in order to complement the data obtained for inhibition of cell fragmentation by VirB-translocated BepE. To this end we used a lentiviral transduction system to generate GFP, GFP-BepE*_Bhe_*, GFP-BIDs.E*_Bhe_* or GFP-BID2.E*_Bhe_*-expressing HUVECs. Lentiviral transduction resulted in mixed cultures of transduced HUVECs expressing the GFP-fusion protein and GFP-negative non-transduced cells. Such mixed cultures were infected with *Bhe* wild-type, *Bhe* Δ*bepE* (Fig. S3) and *Bhe* Δ*bepDEF* (Fig. 6A, B) or left uninfected. Microscopic analysis showed that non-transduced and GFP-positive cells, expressing plain GFP as control, resulted in much higher fraction of HUVECs displaying cell fragmentation than cells expressing GFP-BepE*_Bhe_*, GFP-BIDs.E*_Bhe_* or GFP-BID2.E*_Bhe_* (Fig. 6A and Fig. S3). Expression of the full-length GFP-BepE*_Bhe_* fusion protein and its truncated derivatives in HUVECs were validated by anti-GFP western blot (Fig. S4). Considering that the cell fragmentation at late time points of the infection (48 h) eventually leads to the decrease of the cell number within the sample (Fig. 1B), we used flow cytometry for quantification of this phenotype. To do so, we monitored the ratio of transduced (GFP-positive) vs. non-transduced (GFP-negative) in both infected and uninfected HUVEC populations (Fig. 6B). We considered an increase in the ratio of GFP-positive cells in the infected sample to be indicative of a protective phenotype mediated by the GFP-fusion protein, knowing that cells undergoing fragmentation would be lost during the experiment. These analyses confirmed that the cells expressing GFP-BepE*_Bhe_*, GFP-BID2.E*_Bhe_* and to a lesser extent GFP-BIDs.E*_Bhe_* were protected from fragmentation and toxic effects induced by infection with the BepE-deficient strains, *Bhe* Δ*bepE* and *Bhe* Δ*bepDEF*.

### BepE*_Bhe_* is recruited to the plasma membrane, localizes to cell-to-cell contacts and is enriched in the rear edge of migrating HUVECs

**Figure 7:**
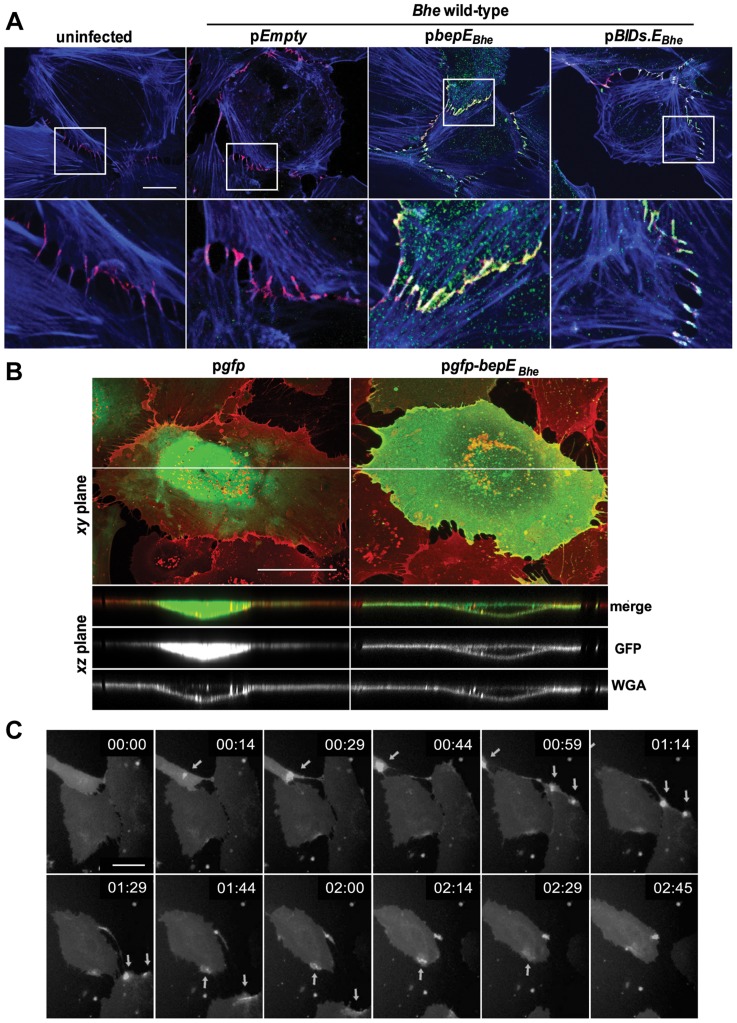
BepE*_Bhe_* localizes to cell-to-cell contacts and is recruited to the plasma membrane of HUVECs following translocation via the T4SS or by ectopic expression. (**A**) Subconfluent monolayers of HUVECs were infected with MOI = 100 of the indicated bacterial strains for 24 h or left uninfected. After fixation and subsequent immunocytochemical staining the specimen was analyzed by confocal laser scanning microscopy. F-actin is represented in blue (Phalloidin) and VE-cadherin staining in red (anti-VE-cadherin). Translocation of the effector protein into the infected cells was detected by anti Myc-staining depicted in green (scale bar = 20 µm). (**B**) HUVECs of an early passage were transduced with lentiviruses directing expression of either GFP or GFP-BepE*_Bhe_*. Cells were stained with wheat germ agglutinin (WGA, red) and fixed. Confocal images were acquired in *xy*- and *xz*-planes (scale bar = 50 µm). (**C**) *gfp-bepE_Bhe_*-transduced HUVECs were subjected to live cell imaging using an MD ImageXpress Micro automated microscope. Snapshots of gray scale images at different time points as depicted by the time stamps (format: dd:hh:mm) are presented (scale bar = 50 µm). The arrows are pointing to the regions of transient enrichments of BepE*_Bhe_* in migrating HUVECs.

In the previous section we showed by anti-Myc immunocytochemistry in combination with confocal microscopy the VirB T4SS-dependent translocation of Myc-BepE*_Bhe_* into HUVECs. Myc-BepE*_Bhe_* localized in a punctuate staining pattern to the plasma membrane, with some enrichment to cell contact areas (Fig. 5A). Due to that latter we performed a co-staining for Myc-BepE or Myc-BIDs.E*_Bhe_* additionally with VE-cadherin, a marker for adherence junctions. The stainings demonstrated co-localization of full length BepE or the BID domains with VE-cadherin (Fig. 7A).

To test for the subcellular localization of GFP-BepE*_Bhe_* ectopically expressed in HUVECs, samples were analyzed by confocal microscopy and images were taken in the *xy*- and the *xz*-planes. GFP-BepE*_Bhe_* clearly localized to plasma membrane with a clear exclusion of signal in the nucleus, while GFP alone localized to the cytoplasm and the nucleus (Fig. 7B). Similarly to the immunostaining of Myc-BepE*_Bhe_*, confocal analysis of ectopically expressed GFP-BepE*_Bhe_* revealed accumulation of BepE at cell-to-cell contacts (Fig. S4, Movie S5).

Further, when GFP-BepE*_Bhe_*-expressing HUVECs were investigated in time-lapse, GFP signal was transiently accumulating and localizing to the rear edge of the cell. As displayed in Movie S6 or the series of images taken at 15 min intervals in Fig. 7C, the GFP signal was increasing at the rear sites of cell detachment, making it conceivable to assume that this transient accumulation of BepE*_Bhe_* may have a role in restoring rear end retraction during cell migration when the process is distorted in cells infected with *Bhe* mutants deficient of BepE.

### Ectopic expression of BepE*_Bhe_* interferes with the disruption of stress fibers by Rho inhibitor I

RhoA is known to be involved in cell body contraction and rear release [28], [29], [30], [31]. Since the fragmentation of HUVECs by infection with *Bhe* Δ*bepDEF* or Δ*bepE* mutant bacteria was associated with a distortion of rear edge detachment during cell migration, and because GFP-BepE*_Bhe_* transiently localizes to this rear edge during detachment, we became interested in testing the role of the RhoA signaling pathway for BepE activity.

**Figure 8:**
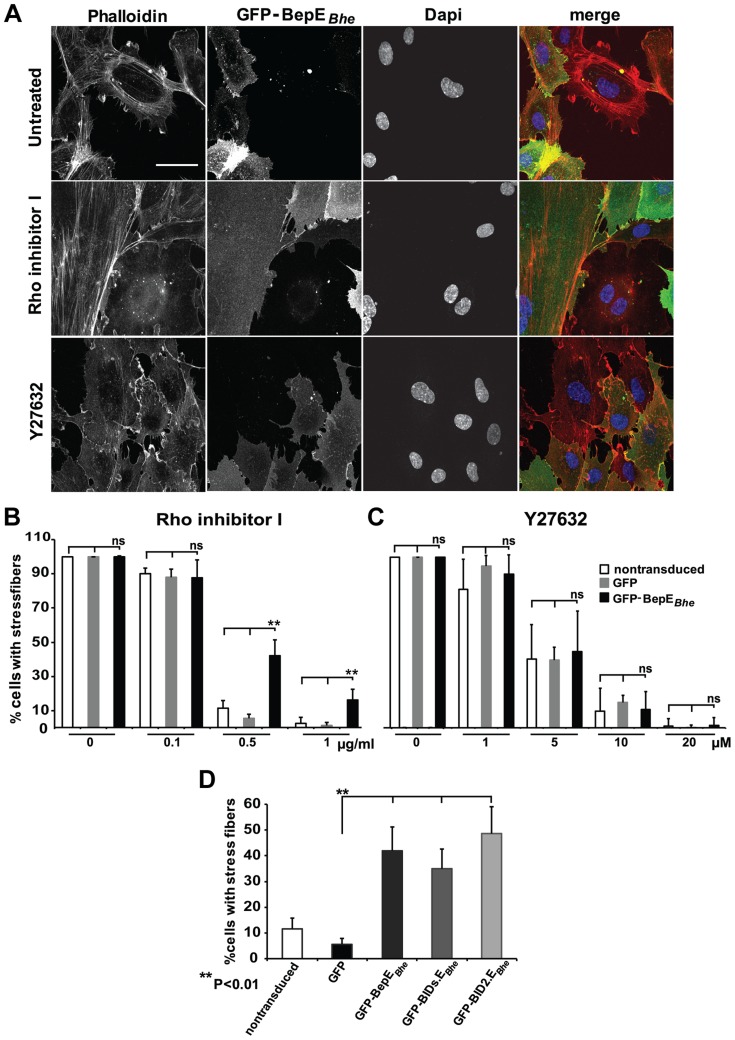
BepE*_Bhe_* interferes with the effect of Rho inhibitor I. (**A**) A mixed population of HUVECs lentivirally transduced with a GFP-BepE*_Bhe_* construct (green) were treated either with 0.5 µg/ml Rho inhibitor I or 10 µM Y27632 (4 h and 30 min respectively) or were left untreated. Cells were fixed, followed by staining for F-actin with Phalloidin (red) and nucleus with DAPI (blue). Representative confocal images are depicted which show selective retainment of actin stress fibers in the GFP-BepE*_Bhe_*-expressing subpopulation (scale bar = 50 µm). (**B**) Dose-dependent effect of Rho inhibitor I on HUVECs expressing GFP-BepE*_Bhe_*. Cells with actin stress fibers in GFP- or GFP-BepE*_Bhe_*-transduced or non-transduced HUVECs were quantified in semi-automated manner, similarly as described for Fig. 1C and D. The percentage of stress fiber-containing cells is shown in the figure. In each condition triplicate wells with each 10 randomly picked microscopic fields were analyzed and represented as mean +/− SD. Statistical significance was determined using Student's *t*-test. *P*<0.05 was considered statistically significant. Data from one representative experiment (n = 3) are presented. (**C**) Dose-dependent effect of Y27632 on HUVECs expressing GFP-BepE*_Bhe_*. Cells with actin stress fibers within GFP or GFP-BepE*_Bhe_*-transduced or non-transduced HUVECs were quantified in semi-automated manner as described for (B). (**D**) Comparison of the potency of GFP-BepE*_Bhe_* and mutant derivatives to interfere with Rho inhibitor I. Lentivirally transduced HUVECs expressing GFP-fusions of BepE*_Bhe_* and the depicted mutant derivatives were treated with 0.5 µg/ml Rho inhibitor I. Specimen were stained and stress fiber-containing cells were quantified as described in (B).

The mixed population of GFP-BepE*_Bhe_*-expressing and non-expressing HUVECs was treated with Rho inhibitor I (CTO4), which is known to inactivate RhoA, B and C by ADP-ribosylation. In response to intoxication by this inhibitor cells initially lose their stress fibers, then round up and eventually die [32], [33], [34]. Interestingly, HUVECs expressing GFP-BepE*_Bhe_* showed resistance to the Rho inhibitor I as shown by the selective preservation of stress fibers, while neighboring non-transduced cells identified by the lack of GFP signal displayed complete resolution of their stress fibers (Fig. 8A). These phenotypes were quantified using HUVECs expressing plain GFP as control sample (Fig. 8B). Interference of GFP-BepE*_Bhe_* with the activity of Rho inhibitor I was dose-dependent (Fig. 8B). Treatment of HUVECs expressing GFP-BepE*_Bhe_* mutants with the same Rho Inhibitor I showed that the second BID domain (BID2.E*_Bhe_*) of BepE is sufficient to interfere with the activity of Rho inhibition (Fig. 8D).

These results support the interaction of BepE with the Rho signaling pathway. To address the question whether BepE acts downstream of ROCK, an effector of RhoA, GFP-BepE*_Bhe_*-transduced HUVECs were exposed to the inhibitor Y27632 that is known to inhibit both ROCK1 and ROCK2 [35]. In contrast to the effect mediated by the Rho inhibitor I, Y27632-treated GFP-BepE*_Bhe_* HUVECs lost the stress fibers similar to the plain GFP-expressing control cells (Fig. 8C). Thus, BepE may have a direct or indirect effect on RhoA or even on ROCK, but not further downstream in this signaling pathway.

### *Btr* Δ*bepDE* loses the ability to colonize rat blood in *intradermal* infection

**Figure 9:**
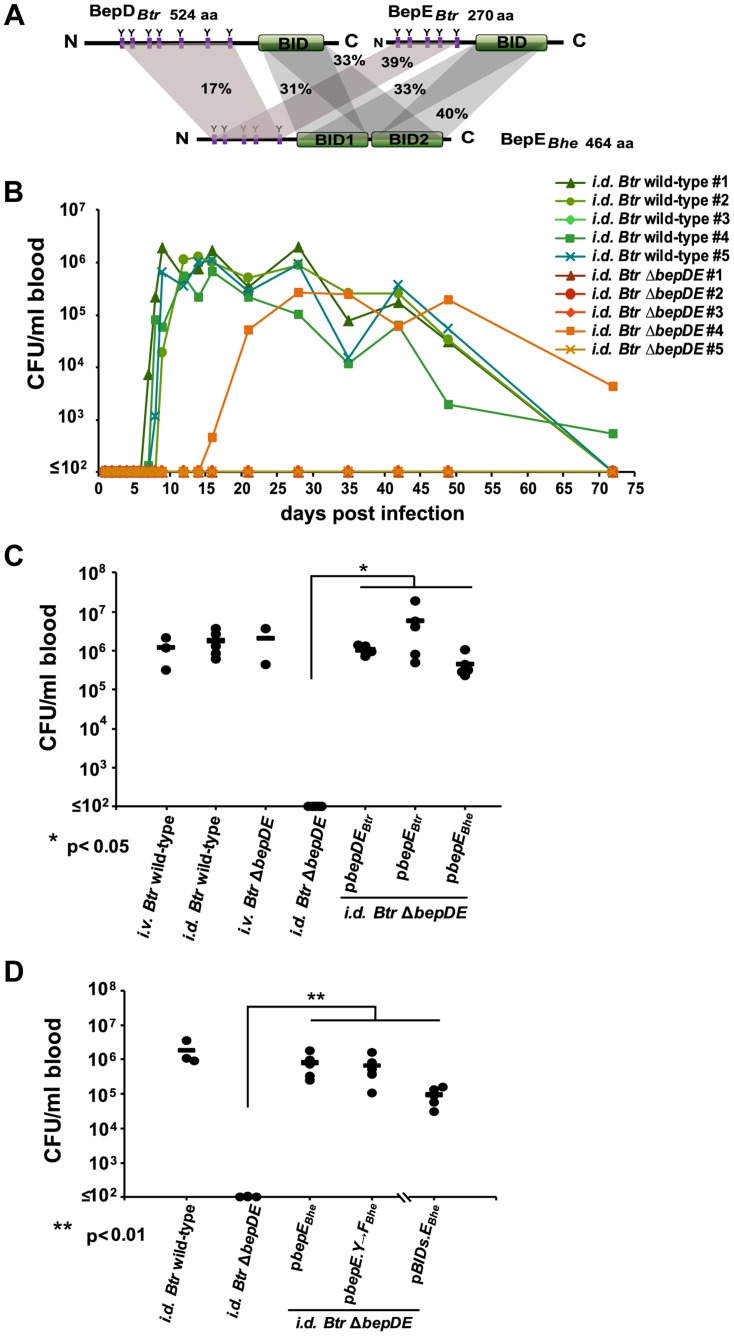
BepE is essential for *Bartonella tribocorum* (*Btr*) to establish bacteremia after *intradermal* (*i.d.*) infection of the rat reservoir host. (**A**) Domain organization of BepE orthologues in *Btr* and *Bhe*. The BepE homologues from *Bartonella* species depicted in the figure (BepE*_Bhe_*, BepD*_Btr_*, BepE*_Btr_*) were aligned using Geneious Pro 5.3.4. The amino acid sequence alignment with pairwise % identity is indicated. The tyrosine-containing N-termini and BID domains were aligned independently. (**B**) *Btr* Δ*bepDE* is not able to reach the blood of rats infected by the *i.d.* route. Rats (n = 5) were inoculated in the ear dermis with either *Btr* wild-type or *Btr* Δ*bepDE*. Blood was drawn at the indicated days post infection (dpi), diluted and plated on sheep blood supplemented Columbia agar plates (CBA) for counting of colony forming units (CFUs). (**C**) Complementation of the *Btr* Δ*bepDE* mutant with BepE is sufficient to restore bacteremia in rats infected by the *i.d.* route. Groups of rats (n≥3) were infected with the indicated strains by the *i.v.* or *i.d.* route. Blood was drawn at 16 dpi and CFUs were recovered as described for B. The graph represents CFUs/ml of blood for individual animals (circles) and their cohort mean (line). Statistical significance was determined using Student's *t*-test. *P*<0.05 was considered statistically significant. (**D**) Heterologous complementation of *Btr* Δ*bepDE* with p*BIDs.E_Bhe_* is sufficient to rescue the abacteremia phenotype following infection by the *i.d.* route. The infections were performed as described for (C). Data represented for BIDs.E*_Bhe_* complementation were acquired in separate experiment from the other data shown. *P*<0.05 was considered statistically significant.

Observing that BepE interferes with deleterious secondary effects of other Beps on migrating cells during *Bartonella* infection *in vitro* we became interested to examine the role of BepE in the establishment of infection *in vivo*. *Bhe* causes intraerythrocytic bacteremia as hallmark of infection in its feline natural reservoir host only, which for ethical reasons is not an accessible experimental model. However, as we have noted functional conservation among BepE homologues (including *Btr*) in protecting the *Bhe* Δ*bepDEF*-mediated fragmentation of HUVECs we reasoned that we can switch to *Btr* as appropriate species for experimental infection of rats as its natural host. Initially we employed the well-described *intravenous* (*i.v.*) infection by *Btr* that leads to the onset of intraerythrocytic bacteremia at 5 days post infection (dpi) following colonization of the so called “primary niche” that is considered to include vascular endothelial cells [14], [17]. *Btr* encodes the BepE*_Bhe_* homologues BepD*_Btr_* and BepE*_Btr_* (Fig. 9A) that both cause partial protection of HUVECs from *Bhe* Δ*bepDEF*–mediated cell fragmentation (see Fig. 3). We thus constructed an in-frame deletion of *bepD* and *bepE* (*Btr* Δ*bepDE*) and compared its course of infection to wild-type bacteria. Rats were injected with *Btr* wild-type or *Btr* Δ*bepDE* through the *i.v.* route and bacteremia was monitored over time as colony forming units (CFUs) per ml of blood. In this model, the *Btr* Δ*bepDE* strain did not show any significant difference in its capacity to cause long-term bacteremia with high bacterial burden (Fig. S5, Fig. 9C). Recently, an *intradermal* (*i.d.*) infection model of *B. birtlesii* (*Bbi*) has been introduced by Marignac *et al* [36], where the bacteria are inoculated in the derma on the ear pinnae of mice. This model more likely reflects the natural route of infection, mimicking the animal scratching an area bitten by the arthropod vector, thereby inoculating derma with infectious bacteria from the arthropod feces. We thus adapted *i.d.* inoculation to our rat model to test for a possible role of BepE at the very early dermal stage of natural infection (Fig. 9B, Fig. S6A). To this end we injected rats with *Btr* wild-type or *Btr* Δ*bepDE* bacteria through the *i.d.* route and monitored the development of bacteremia of infected animals. *Btr* wild-type-infected animals developed blood colonization between 7–9 days post infection (dpi), which occurs with 3–4 day delay as compared to the *i.v.* model (Fig. S6A). In sharp contrast, 12 out of 13 *Btr* Δ*bepDE*- *i.d.* infected rats (from 3 different experiments) stayed abacteremic over 70 days (Fig. 9B). Only one *Btr* Δ*bepDE*-infected animal developed a delayed bacteremia starting on day 16 post infection (Fig. 9B, animal i.d. *Btr* Δ*bepDE* #4), which might result from the fact that the *i.d.* infection protocol can damage capillaries and small vessels in the dermis and thus inoculate bacteria directly into circulating blood similar to the *i.v.* inoculation protocol, yet at much smaller bacterial numbers.

Taken together, the data obtained in the rat infection model suggest that either one of the missing effectors in *Btr* Δ*bepDE*, BepD*_Btr_* or BepE*_Btr_*, or both of them, are of pivotal importance for *Bartonella* to colonize the reservoir host when inoculated in the derma reflecting natural arthropod-borne infection, while the mutant is as infectious as wild-type bacteria via the artificial *i.v.* infection route.

### Expression of BepE in *Btr* Δ*bepDE* restores bacteremia in *i.d.* infected rats

Next we complemented the *Btr* Δ*bepDE* mutant strain with various BepE homologues to test for restoration of bacteremia following *i.d.* inoculation in rats. To this end we expressed *in trans* from a plasmid under control of their native promoter(s) either both BepD*_Btr_* and BepE*_Btr_*, or BepE*_Btr_* alone. Moreover, to test for heterologous complementation we expressed BepE*_Bhe_* in the *Btr* Δ*bepDE* mutant. Following inoculation by the *i.d.* route all complemented mutant strains had developed bacteremia on 16 dpi (Fig. 9C, Fig. S6B).

Taken together these data demonstrate for the *i.d.* rat model that complementation of the abacteremic *Btr* Δ*bepDE* mutant with either BepE*_Btr_* or its homologue BepE*_Bhe_* is sufficient to restore bacteremia. This finding strongly supports a functional conservation of BepE between different species of the genus *Bartonella* that is in line with our *in vitro* studies on the protection from cell fragmentation (see Fig. 3B).

### The BID domains of BepE*_Bhe_* are sufficient to enable *Btr* to reach the blood

Since BepE*_Bhe_* functionally complemented the Δ*bepDE* mutant for bacteremia establishment in the *i.d.* inoculation model we next tested to which domain this activity is confined and whether this might coincide to the BID domains as determined for the protection against cell fragmentation *in vitro* (Fig. 2 and 6). To this end, mutant variants of BepE*_Bhe_* were ectopically expressed in the *Btr* Δ*bepDE* background and tested in the *i.d.* infection model. Two mutants of BepE*_Bhe_* were chosen, one with all five tyrosines exchanged to phenylalanine (Y_37_,_64_,_91_,_106_,_129_→F) and the second, a truncated version of BepE*_Bhe_*, with only the two BID domains and positively charged C-tail (BIDs.E*_Bhe_*). The expression of the BepE*_Bhe_* mutant proteins was confirmed by western blotting (Fig. S6C). At 16 dpi both complemented *Btr* Δ*bepDE* mutants triggered bacteremia at similar levels as the mutant complemented with full-length p*bepE_Bhe_* or as *Btr* wild-type bacteria (Fig. 9D). Thus, the tyrosine-containing motifs of BepE*_Bhe_*, including its N-terminal end, are not required for *Bartonella* to reach its replicative niche in the reservoir host. The functional part of BepE for this infection phenotype can therefore be assigned to the BID domains.

### *Bartonella* is able to translocate the effector protein within dendritic cells and affects their migration

*In vivo* experimental data from rat *i.d.* infections suggests a role for BepE in the establishment of infection at very early stages, prior to the invasion of the blood system. In addition, the *in vitro* model demonstrates that BepE is essential to inhibit fragmentation of infected HUVECs by interfering with the effect of other Beps. To better understand the *in vivo* relevance of BepE and based on our *in vitro* data, we decided to further explore the impact of BepE on cell migration for a cell type potentially relevant to the very early infection process. Assuming that the initial site of *Bartonella* infection is in derma, we speculate that innate immune cells represent the yet unidentified primary infection niche and consider resident dendritic cells (DCs) as a candidate cell type. These cells have high potency of phagocytosis and the property to migrate towards lymph nodes [37], [38], [39].

**Figure 10:**
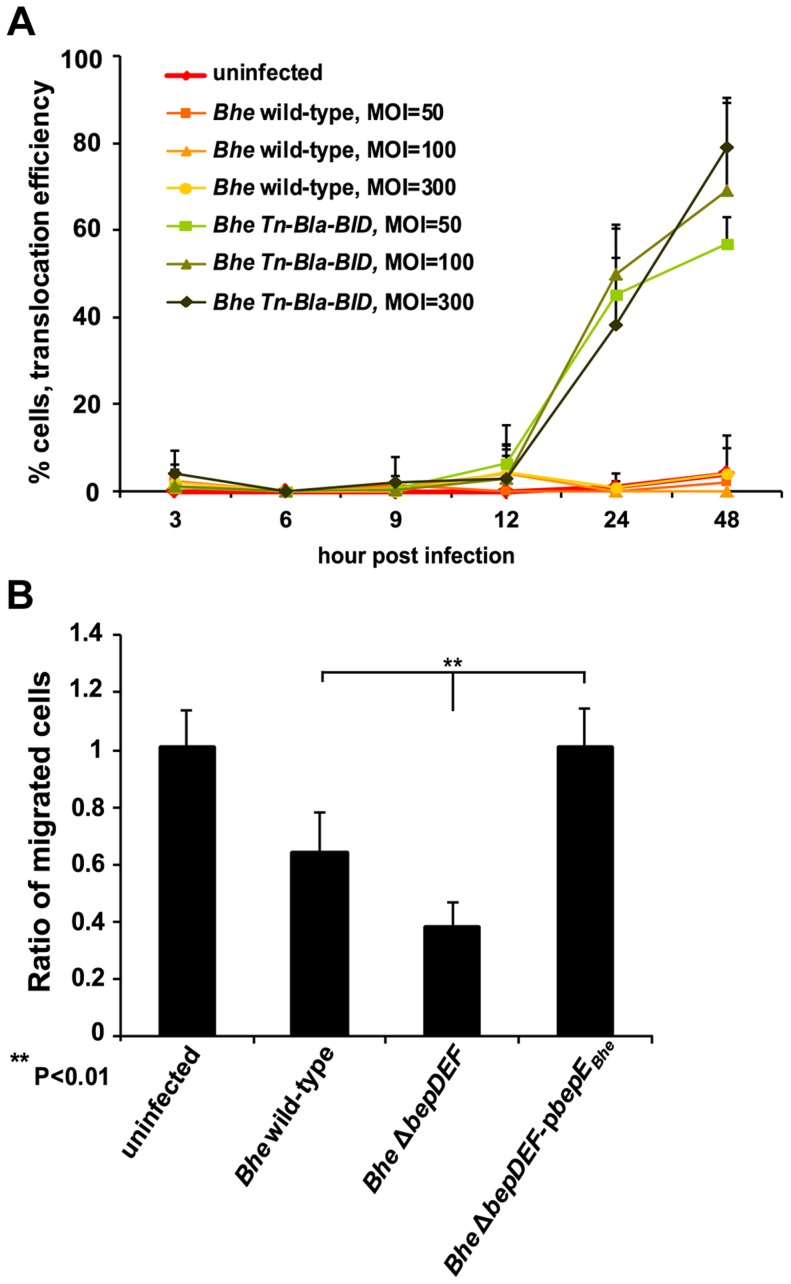
Dendritic cells are infected by *Bartonella*. (**A**) Effector translocation by *Bhe* into mouse bone marrow-derived dendritic cells (BMDCs). Balb/c mouse BMDCs were infected with corresponding MOIs and strains. “Effector”, Bla-BID, translocation efficiency was assessed as the % of infected cells that converted CCF2-AM blue emission into green detected by Leica DM-IRBE inverted fluorescence microscope. The bars represent the mean of triplicate samples +/− SD. Data from one representative experiment (n = 2) are presented. (**B**) Migration of BMDCs is inhibited in a trans-well assay by *Bhe* Δ*bepDEF* infection. BMDCs were pre-infected with MOI = 50 of the indicated bacterial strains. Infected cells were embedded in collagen and mounted in a trans-well migration system that was prior seeded with a confluent monolayer of iLECs (immortalized lymphatic endothelial cells). BMDCs that migrated though the iLECs were quantified after 24 h. The data normalized to uninfected condition. The bars represent the mean of triplicate samples +/− SD. Statistical significance was determined using Student's *t*-test. *P*<0.05 was considered statistically significant. Data from one representative experiment (n = 3) are presented.

First we assessed the effector translocation by *Bhe* into the BMDCs (bone marrow-derived dendritic cells). The strain expressing Bla-BID, a beta-lactamase fusion protein with a bipartite translocation signal from *Bhe* BepD [21], was used to infect mouse BMDCs. β-lactamase activity was measured using CCF2, a FRET substrate that emits green fluorescence but emits blue fluorescence after cleavage by β-lactamase [40]. Starting at 12 hpi the Bla-BID translocation was observed within infected BMDCs (Fig. 10A).

To investigate the migration of *Bartonella*-infected DC towards the draining lymph nodes, we decided to use a trans-well migration assay [41], a tissue culture model system incorporating a 3D extracellular matrix and interstitial flow, where the infected BMDCs have to migrate first through the 3D extracellular collagen matrix and then a layer of iLECs (immortalized lymphatic endothelial cells), thus mimicking the entry of lymphatic system by an infected DC.

As shown in Fig. 10B, BMDCs infected prior with *Bhe* Δ*bepDEF* revealed less efficiency in 3D trans-well migration compared to uninfected or *Bhe* wild-type infection conditions. Similarly to HUVEC cells, the BMDC migration is also affected by *Bhe* Δ*bepDEF* and rescued by BepE. Thus, *in vitro* effector translocation and modulation of cell migration, together with the general relevance of DCs for infection processes, prioritizes these cells for future investigations of the *in vivo* functions of *Bartonella* effector proteins.

## Discussion

Various bacterial pathogens secrete multiple effectors that act in concert to modulate different host cellular functions during the course of infection. Often these effectors may interfere with the activity of each other – either directly or indirectly – in order to orchestrate their multi-pronged interactions with the host in a spatially and temporally controlled manner. We have discovered a particularly striking example in *Bartonella*. We found that BepE acts via BID2.E*_Bhe_* on the RhoA signaling pathway, thereby alleviating deleterious secondary effects of BepC and possibly other Beps. These Beps have distinct functions within the host cell; however, in the absence of BepE, they result in an impaired cell migration and subsequent fragmentation of the infected ECs. Moreover, the rat model of *Btr i.d.* infection, which recapitulates the natural way of *Bartonella* infection by an arthropod, revealed the role of BepE and its BID domains on the dermal stage of infection, thus showing its essential role in establishing reservoir host infection.

*Bartonella* effectors are known to exhibit functional redundancy, i.e. the ability of structurally different elements to perform a similar function [42]. As an example, the F-actin-dependent uptake of *Bhe* via the invasome structure is triggered by distinct Bep-dependent pathways, either by BepC*_Bhe_* and BepF*_Bhe_*, or by BepG*_Bhe_* alone [27]. All Beps harbor at their C-terminus a bipartite T4SS signal composed of a BID domain and a positively-charged tail sequence [21], while only BepD*_Bhe_*, BepE*_Bhe_* and BepF*_Bhe_* contain multiple tyrosinephosphorylation motifs (ITIM/ITSM) in their N-terminal domains [22]. Assuming functional redundancy among this class of effectors we initially focused on the *Bhe* Δ*bepDEF* mutant in infection experiments in HUVECs, the latter representing a well-established *in vitro* model for *Bhe* infection [23], [24], [25], [26], [27]. HUVEC infection with *Bhe* Δ*bepDEF* resulted in a prominently impaired cell migration phenotype. Cell migration is a complex process that requires a series of repetitive but highly coordinated processes [43], [44], [45], [46], [47], [48], [49]. The first step of forward protrusion of lamellipodia and filopodia involves extensive remodeling of the F-actin cytoskeleton. Next, attachment at the leading edge occurs via focal adhesion complexes. After the formation of new adhesions, cells undergo actomyosin-dependent contraction to pull the cell body forward. Coincidently, cell adhesions at the rear of the cell are released such that the cell can move forward [43], [44], [45], [46], [47], [48], [49]. *Bhe* Δ*bepDEF*-infected cells were deficient in rear end detachment and thus underwent fragmentation. Despite the similarity in domain architecture of BepD*_Bhe_*, BepE*_Bhe_* and BepF*_Bhe_*, only BepE*_Bhe_* and its homologues in other *bartonellae* were able to suppress cell fragmentation, which was shown both by bacterial mutant complementation and by ectopic expression of GFP-BepE*_Bhe_* within HUVECs.

The cell fragmentation phenotype is T4SS-dependent and strictly requires translocation of BepC*_Bhe_*, while we have not tested whether other Beps not encoded in the cell fragmentation-inducing strain *Bhe* Δ*bepDEF* (i.e. BepA, BepB or BepG) may also contribute to the cell fragmentation phenotype. As already mentioned, BepC*_Bhe_*, BepF*_Bhe_* and BepG*_Bhe_*, are involved in invasome formation via massive F-actin rearrangements [24], [27]. While this process enables *Bartonella* to be taken up efficiently as large aggregates it is tempting to speculate that the action of BepC and possibly BepG on cell cytoskeleton could affect rear end detachment during cell migration and thereby trigger the cell fragmentation phenotype. Although, by ectopic co-expression with BepC in HUVECs we demonstrated that BepE is reducing the stark influence on a cell cytoskeleton, future studies should address whether BepE acts as a “metaeffector” [50], which acts directly on BepC to modulate its function inside host cells, or rather indirectly tempering its activity at a different level.

GFP-tagged BepE*_Bhe_* was found to be transiently enriched at the rear edge of migrating cells. This localization is consistent with a potential role of BepE in modulating focal adhesion turnover at the rear edge of the cell as a possible mechanism to counteract the cell fragmentation phenotype triggered by other Bep(s). Focal adhesion kinase (FAK), GEFs for Rho and the Rho effectors ROCK and mDia are crucial for the regulation of the adhesion turnover and rear edge retraction [28], [29]. mDia is a formin that produces F-actin filaments by nucleation and polymerization, while ROCK activates myosin to cross-link to F-actin for induction of actomyosin bundles and contractility [51], [52]. Functional interferences with any of these components would disturb normal cell migration. Small GTPases are targeted by numerous bacterial effectors and several examples are known for pairs of effectors secreted by a given pathogen that have opposing effects on their regulation. *Salmonella* SopE and SptP have adopted GEF and GAP functions for CDC42 and RAC1, respectively, that allow to temporally switch between their active and inactive forms; first to induce membrane ruffle-mediated uptake of bacteria, which is followed by stabilization of the remodeled F-actin cytoskeleton [53]. Another example is the *Legionella pneumophila* effectors Drra/SidM and LepB that are known to regulate the Rab1 activity and dynamic membrane remodeling of the *Legionella*-containing phagosome [54]. The Bep-dependent cell fragmentation phenotype, observed in the absence of BepE, might be related to the inhibition of RhoA. Inhibition of RhoA activity by its inhibitor, C3 (*C. botulinum* exoenzyme), leads to marked morphological changes of migrating monocytes, with frequent polarization and long tails trailed behind the cell body [30] - a phenotype reminiscent to an early stage of cell fragmentation. A counteraction of BepE on the RhoA signaling pathway is supported by the fact that ectopic expression of GFP-BepE*_Bhe_* interferes with the inhibitory effect of the C3-based Rho inhibitor 1, as shown by stress fiber preservation in GFP-BepE*_Bhe_*-expressing cells. However, BepE was not able to potentiate any stress fibers when using the ROCK inhibitor Y27632. Based on these observations, BepE could be a factor that directly (or indirectly) activates RhoA independently of Rho inhibitor 1-mediated ADP-ribosylation at asparagine 41 [33].

The VirB T4SSs of *bartonellae* and its multiple Bep effectors evolved as a toolbox facilitating the adaptation to specific mammalian reservoir hosts [15], [55]. The prominent activity of BepE homologues in restoring cell migration raised our interest to decipher the *in vivo* function of this effector protein. *Bartonella*-reservoir host interaction has been studied in a rat model developed by Schulein *et al* [14]. In this model, intravenously (*i.v.*) injected *Btr* wild-type are rapidly cleared from the blood stream, which remains sterile for at least three days. On day four, the bacteria re-appear in the blood where they adhere to and invade erythrocytes. Approximately every five days, a new wave of bacteria is seeded into the blood stream and invades erythrocytes, sustaining the bacteremia for about ten weeks. This is in accordance with other animal models, such as *B. grahamii* (*Bgr*) *i.v.* infection of mice [56]. The intimate interaction of *bartonellae* with ECs *in vitro* and the clinical descriptions of *Bhe* association to human vasculature, together with the disappearance of bacteria from blood for several days following *i.v.* infection lead to the proposition that vascular ECs represent, at least in part, the “primary niche” that is colonized initially and from where bacteria are seeded to the blood stream to invade erythrocytes. Upon *i.v.* infection the course of bacteremia was similar in cases of wild-type and *Btr* Δ*bepDE* mutant bacteria. In an *i.d.* infection model that reflects much better the initial dermal stage of *Bartonella* infection, wild-type bacteria still caused bacteremia, yet with a 4–5 day time delay compared to the *i.v.* model. In contrast, animals infected with the *Btr* Δ*bepDE* mutant remained abacteremic, while complementation with BepE restored bacteremia. This data suggests (i) that *Bartonella* presumably initially infects some cell type(s) in the derma, and (ii) that spreading the infection to the blood stream (possibly via the lymphatic system) is BepE-dependent. Previous studies based on the *i.v.* infection model describe components of *Btr* VirB T4SS to be essential for colonization of the “primary niche”, while those were dispensable for the subsequent stage of intraerythrocytic infection [17], [19]. Addressing an earlier stage of vector-borne infection, our results in the *i.d.* model demonstrate that the VirB T4SS and specifically BepE are already required at the dermal stage of infection. This revisits our understanding of intracellular niches of *Bartonella*. We suggest replacing the old term “primary niche” by “blood seeding niche” and introducing “dermal niche” as a new term describing the preceding dermal stage of arthropod-borne infection.

The BepE-dependent phenotypes *in vivo* (progression from dermal to blood stage infection in the *i.d.* model) and *in vitro* (suppression of cell fragmentation and stress fiber disassembly by Rho inhibitor 1 activity) were consistently mediated by the BID domains of BepE*_Bhe_*. In fact, most phenotypes mediated by Beps of *Bhe* are attributed to a BID domain – further to the essential role of the most C-terminal BID domain as part of the bi-partite secretion signal for T4SS-dependent effector translocation [21] this domain has thus adopted diverse effector functions within host cells [15]. The anti-apoptotic activity of BepA*_Bhe_* is confined to its single BID domain [27], and the F-actin remodeling activity of BepF is mediated by its multiple BID domains. Surprisingly, the phosphotyrosine-containing motifs in the N-terminal part of BepE that were shown to recruit multiple SH2-domain containing signaling proteins of the host cell [22] revealed no essential role in the here described BepE-dependent *in vitro* and *in vivo* phenotypes. However, we suggest that investigation of these motifs may be relevant in cells infected in the “dermal niche”, e.g. to modulate immune responses. DCs represent one of the candidate cell types for *Bartonella* infection in this “dermal niche”. In steady state skin DCs include immature epidermal DCs, Langerhans cells (LCs) and immature dermal DCs [57]. Those have a high phagocytic activity and constantly sample the environment as sentinels for foreign antigens (Ag) [58]. Ag uptake induces the activation/maturation of DCs by starting a directional migration towards the draining lymph nodes and up-regulation of MHC class II/co-stimulatory molecules. Finally, in the lymph nodes DCs form an immune synapse with naive T cells and present Ags, and as a consequence initiate effective adaptive immune responses [59], [60]. Considering that BepE is recruited to cell-to-cell contacts, the phosphotyrosine motifs and their proposed mimickry of immune inhibitory receptors [22] could be a valid tool to modulate signaling pathways within the immune synapse and thus altering the initiation of an adaptive immunity. On the way to lymph nodes DCs may serve as transporters of bacterial cargo. This strategy is considered to be used by other bacterial pathogens, such as *Brucella abortus* [61] or *Bordetella bronchiseptica* [39], to disseminate within their mammalian hosts. When *Btr* Δ*bepDE* infects the rat reservoir host we think that at the dermal stage fragmentation or impaired migration of yet unknown infected cells may occur that would prevent further dissemination of bacteria and colonization of the blood stream. This is further supported by our finding that migration of BMDCs in a trans-well migration assay is blocked by infection with *Bhe* Δ*bepDEF*, while expression of BepE*_Bhe_* in this mutant background restores it. Albeit it is difficult to compare directly the phenotypic changes in DC (altered cell migration) and HUVECs (fragmentation) upon *Bartonella* infection, yet considering dermal DCs as “dermal niche” for *Bartonella* represents a valid hypothesis to be investigated *in vivo*. Future work should also address how exactly the complex *in vitro* phenotype of cell fragmentation translates into the striking *in vivo* phenotype - lack of spreading from derma to blood.

In summary, this study builds on extending the “primary niche” of *Bartonella* infection that precedes blood stage infection in the mammalian reservoir host to a dermal stage reflecting natural infection by blood-sucking arthropods. Using this *i.d.* model we describe for the first time a specific role for a *Bartonella* effector protein *in vivo*, i.e. BepE is required to spread infection from the dermal site of inoculation to the blood, possibly via promoting normal migration of infected DCs by alleviating deleterious secondary effects mediated by other Beps targeting the RhoA pathway. As a result, this functional crosstalk between Beps could facilitate that *Bartonella* reaches its replicative niche in the blood, where it multiplies and persists within the immune-privileged intracellular compartment of erythrocytes, from where it is picked up by blood-sucking arthropods for transmission to naive hosts.

## Materials and Methods

### Ethics statement

Animals were handled in strict accordance with good animal practice as defined by the regulations from the Federal Veterinary Office (FVO) of Switzerland: Animal Welfare Act (TSchG;45), Animal Welfare Ordinance (TSchV;455.1) and Animal Experimentation Ordinance (Tierversuchs-V;455.163). Animal work was approved by the Veterinary Office of the Canton Basel City, Kantonales Veterinäramt, on January 2011 (licence no. 1741). Analyses of human umbilical cord endothelial cells (HUVECs) were performed with written informed consent of patients from Bruderholzspital Basel under approval from the Local Research Ethics Committee, Ethikkommission beider Basel EKBB (protocol no. 102/01). All patient samples were anonymized.

### Bacterial strains and growth conditions

The bacterial strains used in this study are listed in Table S1. *Bartonella spp* were grown on Columbia agar plates containing 5% defibrinated sheep blood (CBA plates) at 35°C and 5% CO_2_ for 2–3 days. *Bhe* RSE247 [26] and *Btr* RSE149 [14] spontaneous streptomycin-resistant strains served as *Bhe* wild-type and *Btr* wild-type respectively in this study. When indicated, media were supplemented with 30 µg/ml kanamycin, 100 µg/ml streptomycin, 10 µg/ml gentamycin, and/or 500 µM isopropyl-β-D-thiogalactoside (IPTG, Applichem). *E. coli* strains were cultivated in Luria-Bertani liquid medium (LB) or on Luria-Bertani agar on plates (LA) at 37°C overnight. When indicated, media were supplemented with 50 µg/ml kanamycin, 200 µg/ml ampicillin, 20 µg/ml gentamycin, 500 µM IPTG, and/or 1 mM diaminopimelic acid (DAP).

### Conjugation of *Bartonella*-expression plasmids into *Bartonella*

Conjugation of plasmids from the *E. coli dap*– mutant b2150 [26] into *Bartonella* spp. was done by triparental mating in the presence of helper plasmid pRK2013 as described previously by Dehio *et al.*, 1998 [62].

### DNA manipulations

Plasmids used in this study are listed in Table S1, primers are listed in Table S2. Generation of plasmids for expression of Myc-Beps or lentiviral expression, plasmids used for chromosomal integrations and in-frame deletions are detailed described in Materials and Methods S1.

### Western blot analysis of protein expression in *Bartonella*

Western blot analysis of expressed protein levels in *Bartonella* was performed as described previously by Schulein *et al.*, 2005 [21].

### Infection of rats

Female Wistar rats were obtained at the age of 10 weeks from Harlan, RCC-Füllinsdorf. All animal studies were approved by the authors' institutional review boards.

After two weeks of adaptation the rats were infected with *Btr*. Bacterial strains were grown as described above, harvested in phosphate-buffered saline (PBS), and diluted to OD_595_ = 1. Rats were anesthetized with a 2–3% Isoflurane/O_2_ mixture and infected with 10 µl or 200 µl of the bacterial suspension in the dermis of the right ear or in the tail vein respectively. Blood samples were taken at the tail vein and immediately mixed with PBS containing 3.8% sodium-citrate to avoid coagulation. After freezing to −70°C and subsequent thawing, undiluted and diluted blood samples were plated on Columbia agar plates containing 5% defibrinated sheep blood (CBA plates) at 35°C and 5%. CFUs were counted after 7–10 days of growth.

### Cell culture

Human umbilical vein endothelial cells (HUVEC) were isolated as described before [26] and cultured in EGM medium (Promocell) in a humidified atmosphere at 37°C and 5% CO2.

Mouse bone marrow-derived dendritic cells (BMDCs) were differentiated *in vitro* using standard protocol [63]. Briefly, bone marrow cells were flushed from the tibias and femurs of Balb/c mouse with culture medium composed of DMEM medium (Invitrogen Life Technologies) supplemented with 10% fetal calf serum (FCS, Invitrogen Life Technologies). After one centrifugation, BM cells were resuspended in Tris-ammonium chloride for 2 min to lyse RBC. After one more centrifugation, BM cells were cultured for 9–10 days at 1*10^6^ cells/ml in culture medium supplemented with 200 ng/ml recombinant human Flt3L (produced by 40E1 hybridoma cells, kind gift from prof. A. Rolink). Cultures were incubated at 37°C in 5% CO_2_-humidified atmosphere.

iLECs (immortalized lymphatic endothelial cells) were cultured as described by Vigl *et al.*, 2011 [64].

### Lentiviral transduction of HUVECs

Subconfluent (3*10^6^) HEK 293T cells in 10 cm cell-culture dishes were transfected with a total of 5 µg of plasmid DNA following the FuGENE transfection protocol (FuGENE 6 Transfection Reagent, Roche). After 12 h, the cell culture media was replaced and the cells were kept in culture for virus production in the supernatant for additional 24 h. 7*10^4^ HUVECs/well was seeded in gelatin coated 6-well plate 24 h before the viral infection. On day of infection the viral supernatant from transfected HEK 293T cells was filtered with 0.45 µm filter and transferred onto the monolayer of HUVECs, 2 ml of viral supernatant in presence of 0.5 µg/ml Polybrene (Sigma) was applied on each well. After the first harvesting of viral supernatant the HEK 293T cells were supplemented with a fresh medium. 24 h later the HUVECs were re-infected repeating the same procedure with followed replacement of the medium in 24 h.

### Infection of HUVECs

HUVECs (passage 4–7) were plated on gelatin-coated glass slides in 24-well plates at 5*10^4^/ml using EGM or alternatively 3*10^3^/well seeded in 96-well plate. The next day cells were washed twice with M199 with Earls salts (M199, Gibco, Invitrogen Life Technologies) supplemented with 10% FCS (Invitrogen Life Technologies) and infected with a multiplicity of infection (MOI) of 200 bacteria per cell or in case of mixed infections 200 plus 200 of corresponding strains in M199/10% FCS/500 µM IPTG and incubated for 24, 36 and 48 h or 72 h for the time-lapse microscopy. Where necessary the infection was fixed with 3,7% paraformaldehyde (PFA).

### Immunofluorescent labeling

Indirect immunofluorescent labeling was performed as described [26]. Standard 96-well plate assays were stained with TRITC-Phalloidin (Sigma, 100 µg/ml stock solution, final concentration 1∶400) or Dy-647-Phalloidin (Dyomics, 100 µg/ml stock solution, final concentration 1∶200) and DAPI (Roche, 0.1 mg/ml). Samples on glass slides for confocal microscopy were stained with Cy3-Phalloidin (Sigma, 100 µg/ml stock solution, final concentration 1∶100) or Dy-647-Phalloidin (Dyomics, 100 µg/ml stock solution, final concentration 1∶200), DAPI and anti-Myc antibodies (mouse anti-Myc, Invitrogen, Carlsbad, CA/USA) and/or VE-Cadherin (rabbit anti-VE-Cadherin, Bender MedSystems, Burlingame, CA/USA). Secondary antibodies for Immunofluorescence used in this study were Cy5-conjugated goat anti-rabbit Ig antibodies (Dianova, Hamburg, Germany, 1∶100).

### Inhibitor treatment of transduced HUVECs

Mixed culture of non-transduced and lentivirally transduced HUVECs by pRO300, pRO301, pRO302, pRO303 or pRO304 were seeded in gelatin coated wells at 3,5*10^3^/100 µl using endothelial complete medium (PromoCell) in 96 well-plates in the morning. Cells were starved in endothelial medium with reduced serum (containing 20× reduced SupplementMix) for overnight. The next day the wells were treated with Rho Inhibitor I (CTO4, Cytoskeleton, Denver, CO) or Y27632 (Sigma) at different concentrations for 4 h or 30 m respectively. The experiment was stopped by fixation with 3.7% PFA, stained for F-actin and nucleus as described above and subjected to automated microscopy.

### Image analysis and quantification of cell fragmentation or loss of stress fibers

Experiments performed in 96-well plates were subjected to automated microscopy, using MD ImageXpress Micro automated microscopes. In every well, 6×6 sites were imaged in 2–3 different wavelengths corresponding the applied cell staining. Images were visualized using MetaXpress software (MDC) and the number of cells per image was determined automatically by MetaXpress in-build analysis modules (CountNuclei). The cells with the thin elongations or stress fiber-containing cells were defined and counted by eye. The percentage of elongated cells or stress fiber-containing cells was calculated using Microsoft Office Excel. In every condition randomly picked 10 fields of magnification 10 within the well and triplicates of the wells were analyzed.

For confocal laser microscopy, the stained samples were analyzed using a Zeis Point Scanning Confocal, LSM700 Upright microscope (Imaging Core Facility, Biozentrum, University of Basel, Switzerland). Z-stacks with 20–30 focal planes with a spacing of 0.1–0.3 µm were recorded and xz- and yz-planes were reconstructed using Zen 2010 software. Images were exported and finalized using MetaMorph, ImageJ and Adobe Photoshop.

### Time-lapse microscopy

Time-lapse microscopy was carried out using an MD ImagXpress Micro automated microscope from Molecular devices (inside a controlled chamber held at 37°C and 5% CO_2_). Images were recorded using a CoolSNAP ES digital CCD camera and processed using MetaXpress software. Briefly, HUVECs were infected with *Bhe* strains in 96-well format as described above. After incubating for 8 h, 2 to 4 points in each well were chosen for time-lapse microscopy. The points were imaged for 72 h with 10 min lapse between each imaging. Both, red and green, fluorescence were detected at 20× magnification, and the corresponding videos were processed in MetaXpress and compiled into QuickTime (Apple, Cupertino, CA) movies.

### Quantification of cell survival by FACS analysis

Mixed population of non-transduced HUVECs with lentivirally transduced HUVECs by pRO300, pRO301, pRO302, pRO303 or pRO304 were seeded in gelatin coted 6 well-plates at 10^5^ cell/well the evening before experiment. Next day the infection was performed with MOI 200 as described above. After 48 hpi the cells were detached from the wells by using trypsin, resuspended in medium and analyzed for quantification of GFP-expressing cells by FACS (BD Biosciences). The data were analyzed using the FlowJo software.

### Infection and Bla analysis of mouse BMDCs

Day 9–10 BMDCs were harvested from the flask by vigorous pipetting followed by washing twice with room temperature PBS without Ca^2+^ or Mg^2+^ (Invitrogen Life Technologies). 4*10^3^ BMDCs were seeded within the wells of 96-well plate. Next day supernatant was replaced with the fresh M199 with Earls salts (M199, Gibco, Invitrogen Life Technologies) supplemented with 10% FCS (Invitrogen Life Technologies) and infected with a MOI of 50, 100, 300. At 3, 6, 9, 12, 24 and 48 h post infection the wells were loaded with 1 µM CCF2- AM (Invitrogen Life Technologies) in M199/10% FCS and incubated at room temperature for 60–120 min. The plate was imaged in two channels (blue and green) by Leica DM-IRBE inverted fluorescence microscope. Data were analyzed in MetaMorph and further processed in Microsoft Office Excel.

### BMDC trans-well migration

Trans-well migration assay was performed as described previously [41]. Experiments were performed with 12 mm diameter, 8 µm pore cell-culture inserts (Millipore, Billerica, MA) in a modified Boyden chamber assay. One day before the experiment, iLECs were seeded onto the collagen-coated underside of the chamber at 10^5^ cells/well and in parallel 2*10^5^ mouse BMDCs were infected with *Bartonella* at MOI = 200. The next day, infected BMDCs were harvested and stained with DiD-Vybrant (Invitrogen Life Technologies), then seeded in 200 µl (1 mm thick) Matrigel (4.65 mg/ml, BD Biosciences, San Jose, CA) within the inserts. Basal medium was placed in the top and bottom chamber. After 15 h in a 35°C/5% CO_2_ incubator, Matrigel containing non-migrated cells was removed and the inserts were fixed in 3.7% PFA. The number of migrated cells was counted by microscope on the underside of the chamber.

### Accession numbers for the proteins used in this study

The Uniport (http://www.uniprot.org) accession numbers for the protein sequences to BepA*_Bhe_* (Q6G2A9), BepC*_Bhe_* (Q5QT03), BepD*_Bhe_* (Q5QT02), BepE*_Bhe_* (Q5QT01), BepF*_Bhe_* (Q5QT00), BepG*_Bhe_* (Q5QSZ9), BepD*_Btr_* (A9IWP9), BepE*_Btr_* (A9IWQ0), BepE*_Bqu_* (Q6FYV7), BepH*_Bgr_* (C6AES9), VirB4*_Bhe_* (Q9R2W4).

## Supporting Information

Figure S1 **(A) Sequence alignment of BID1 (BID1.E** ***_Bhe_*** **) and BID2 (BID2.E** ***_Bhe_*** **) domains of BepE** ***_Bhe_*** **.** The BepE BID domains were aligned using Geneious Pro 5.3.4. Identical aa are highlighted in red, similar aa in pink, and non-conserved aa in grey. (**B**) Domain organization of BepE orthologues in *Btr*, *Bhe, Bqu* and *Bgr*. The BepE homologues from *Bartonella* species depicted in the figure (BepE*_Bhe_*, BepD*_Btr_*, BepE*_Btr_*, BepE*_Bqu_* and BepH*_Bgr_*) were aligned using Geneious Pro 5.3.4. The amino acid sequence alignment with pairwise % identity is indicated. The tyrosine-containing N-termini and BID domains were aligned independently. (**C**) Protein levels of the BepE*_Bhe_* homologues, BepE*_Bqu_*, BepE*_Btr_*, BepD*_Btr_* and BepH*_Bgr_* by overexpression in *Bhe* Δ*bepE* and *Bhe* Δ*bepDEF*. The anti-Flag and anti-Myc western blots were obtained from total lysate of corresponding *Bhe* strains.
(TIF)

Figure S2 **BepE inhibits host cell fragmentation upon translocation via T4SS.** Subconfluent monolayers of HUVECs were infected with MOI = 200 or MOI = 200+200 in case of mixed infection depicted in the figure. Quantification of cell fragmentation at 48 h post infection was performed as described for Fig. 1C and D and presented as mean of triplicate samples +/− SD. Statistical significance was determined using Student's *t*-test. *P*<0.05 was considered statistically significant. Data from one representative experiment (n = 2) are presented.
(TIF)

Figure S3 **Ectopic expression of BepE** ***_Bhe_*** **protects HUVECs from cell fragmentation.** (**A, B**) HUVECs of an early passage were transduced with lentiviruses for the expression of the depicted GFP-fusion proteins. The mixed culture of transduced and non-transduced cells were infected with the indicated *Bhe* strains (MOI = 200). Infected cells were either fixed and stained for microscopy or analyzed for the survival by FACS at 48 hpi. (**A**) Representative microscopy images (scale bar = 100 µm). F-actin is represented in red (Phalloidin), DNA in blue (DAPI), GFP in green. (**B**) Protection by GFP-fused BepE and its derivatives against fragmentation induced by *Bhe* strains. GFP-positive cell were quantified by FACS and normalized to the uninfected cell population. One representative experiment (n = 3) with the mean of triplicate samples +/− SD are presented. Statistical significance was determined using Student's *t*-test. *P*<0.05 was considered statistically significant.
(TIF)

Figure S4 **Ectopically expressed GFP-BepE** ***_Bhe_*** **localizes to cell-to-cell contacts.** (**A**) (**B**) HUVECs of an early passage were transduced with lentiviruses directing expression of GFP-BepE*_Bhe_*. Cells were fixed and stained by Phalloidin (Scale bar = 50 µm) (**A**) or stained by wheat germ agglutinin (WGA) and fixed afterwards (Scale bar = 25 µm) (**B**). Samples were subjected to confocal microscopy. (**C**) Lentivirally transduced HUVECs were tested for the expression of respective GFP-fusion proteins. Total-cell extracts were separated by SDS-PAGE and blotted with anti-GFP antibodies.
(TIF)

Figure S5 **Rat** ***intravenous*** **(** ***i.v*** **) infection by** ***Btr*** **Δ** ***bepDE*** **.** Groups of rats (n = 3) were injected in the tail vein (*i.v.*) with the depicted *Btr* strains. Blood was drawn at seven dpi, diluted and plated on sheep blood supplemented Columbia agar plates (CBA) for counting colony forming units (CFU). The graph represents CFUs/ml of blood for individual animals (circles) and their cohort mean (line).
(TIF)

Figure S6 **(A) Comparison of rat blood colonization by** ***Btr*** **wild-type after** ***intradermal*** **(** ***i.d.*** **) and** ***intravenous*** **(** ***i.v.*** **) infections.** Groups of rats (n = 5) were injected in the tail vein (*i.v.*) or in the ear dermis (*i.d.*) with *Btr* wild-type. Blood was drawn, diluted and plated on sheep blood supplemented Columbia agar plates (CBA) for counting of colony forming units. Bacteremia (per ml of blood) of *Btr* wild-type *i.d.*-infected single animals is compared to the *i.v*. infection (dashed line, mean of 6 animals) described previously by Schulein *et al*, 2001. (**B**) Complementation of the *Btr* Δ*bepDE* mutant with BepE is sufficient to restore bacteremia in rats infected by the *i.d.* route. Groups of rats (n≥3) were infected with the indicated strains by the *i.v.* or *i.d.* route. Blood was drawn at 10 dpi and CFUs were recovered as described for (A). The graph represents CFUs/ml of blood for individual animals (circles) and their cohort average (line). Statistical significance was determined using Student's *t*-test. *P*<0.05 was considered statistically significant. (**C**) Protein levels of the BepE*_Btr_* homologue, BepE*_Bhe_* and its mutants by overexpression in *Btr* Δ*bepDE*. The anti-Myc western blots were obtained from total lysate of corresponding *Bartonella* strains.
(TIF)

Table S1 **Bacterial strains and plasmids used in this study.**
(DOCX)

Table S2 **Oligonucleotides used in this study.**
(DOCX)

Materials and Methods S1 **Description of DNA manipulations.**
(DOCX)

Movie S1 **Cell fragmentation induced by** ***Bhe*** **Δ** ***bepDEF*** **and complementation by BepE** ***_Bhe_*** **.** HUVECs expressing LifeAct-mCherry were infected with *Bhe* Δ*bepDEF* or *Bhe* Δ*bepDEF*-p*bepE* and subjected to live cell imaging with an MD ImageXpress Micro automated microscope.
(MOV)

Movie S2 **Random migration of HUVECs infected with** ***Bhe*** **wild-type.** HUVECs expressing LifeAct-mCherry were left uninfected or infected with *Bhe* wild-type expressing eGFP and subjected to live cell imaging with an MD ImageXpress Micro automated microscope.
(MOV)

Movie S3 **Cell fragmentation induced by** ***Bhe*** **Δ** ***bepE*** **and complementation by BepE** ***_Bhe_*** **.** HUVECs expressing LifeAct-mCherry were infected with *Bhe* Δ*bepE* or *Bhe* Δ*bepE*-p*bepE* and subjected to live cell imaging with an MD ImageXpress Micro automated microscope.
(MOV)

Movie S4 **BepE** ***_Bhe_*** **counteracts the effect of BepC** ***_Bhe_*** **.** HUVECs were double transduced for the expression of GFP-BepE*_Bhe_* and mCherry-BepC*_Bhe_* and subjected to live cell imaging with an MD ImageXpress Micro automated microscope.
(AVI)

Movie S5 **GFP-BepE** ***_Bhe_*** **accumulation to cell-to-cell contacts in HUVECs.** HUVECs expressing GFP-BepE*_Bhe_* were subjected to confocal microscopy. Focal planes with a spacing of 0.15 µm were recorded. 3D projection of z stacks is presented.
(AVI)

Movie S6 **Transient accumulation of GFP-BepE** ***_Bhe_*** **in the rear of migrating HUVECs.** HUVECs expressing GFP-BepE*_Bhe_* were subjected to live cell imaging with an MD ImageXpress Micro automated microscope. The arrows are pointing to the regions of transient enrichments of BepE*_Bhe_* localization in randomly migrating HUVECs.
(MOV)

## References

[1] Monack DM, Mueller A, Falkow S (2004) Persistent bacterial infections: the interface of the pathogen and the host immune system. Nat Rev Microbiol 2: 747–765. 15372085 10.1038/nrmicro955

[2] Lee KS, Kalantzis A, Jackson CB, O'Connor L, Murata-Kamiya N, et al (2012) Helicobacter pylori CagA triggers expression of the bactericidal lectin REG3gamma via gastric STAT3 activation. PLoS One 7: e30786. 22312430 10.1371/journal.pone.0030786 PMC3270022

[3] Kido M, Watanabe N, Aoki N, Iwamoto S, Nishiura H, et al (2011) Dual roles of CagA protein in Helicobacterpylori-induced chronic gastritis in mice. Biochem Biophys Res Commun 412: 266–272. 21820415 10.1016/j.bbrc.2011.07.081

[4] Schroeder GN, Hilbi H (2008) Molecular pathogenesis of Shigella spp.: controlling host cell signaling, invasion, and death by type III secretion. Clin Microbiol Rev 21: 134–156. 18202440 10.1128/CMR.00032-07 PMC2223840

[5] Diacovich L, Gorvel JP (2010) Bacterial manipulation of innate immunity to promote infection. Nat Rev Microbiol 8: 117–128. 20075926 10.1038/nrmicro2295

[6] Shames SR, Finlay BB (2012) Bacterial effector interplay: a new way to view effector function. Trends Microbiol 20: 214–219. 22425230 10.1016/j.tim.2012.02.007

[7] Chomel BB, Boulouis HJ, Maruyama S, Breitschwerdt EB (2006) Bartonella spp. in pets and effect on human health. Emerg Infect Dis 12: 389–394. 16704774 10.3201/eid1203.050931 PMC3291446

[8] Boulouis HJ, Chang CC, Henn JB, Kasten RW, Chomel BB (2005) Factors associated with the rapid emergence of zoonotic Bartonella infections. Vet Res 36: 383–410. 15845231 10.1051/vetres:2005009

[9] Chomel BB, Boulouis HJ, Breitschwerdt EB, Kasten RW, Vayssier-Taussat M, et al (2009) Ecological fitness and strategies of adaptation of Bartonella species to their hosts and vectors. Vet Res 40: 29. 19284965 10.1051/vetres/2009011 PMC2695021

[10] Breitschwerdt EB, Maggi RG, Chomel BB, Lappin MR (2010) Bartonellosis: an emerging infectious disease of zoonotic importance to animals and human beings. J Vet Emerg Crit Care (San Antonio) 20: 8–30. 20230432 10.1111/j.1476-4431.2009.00496.x

[11] Dehio C (2004) Molecular and cellular basis of bartonella pathogenesis. Annu Rev Microbiol 58: 365–390. 15487942 10.1146/annurev.micro.58.030603.123700

[12] Foil L, Andress E, Freeland RL, Roy AF, Rutledge R, et al (1998) Experimental infection of domestic cats with Bartonella henselae by inoculation of Ctenocephalides felis (Siphonaptera: Pulicidae) feces. J Med Entomol 35: 625–628. 9775583 10.1093/jmedent/35.5.625

[13] Raoult D, Aboudharam G, Crubezy E, Larrouy G, Ludes B, et al (2000) Molecular identification by “suicide PCR” of Yersinia pestis as the agent of medieval black death. Proc Natl Acad Sci U S A 97: 12800–12803. 11058154 10.1073/pnas.220225197 PMC18844

[14] Schulein R, Seubert A, Gille C, Lanz C, Hansmann Y, et al (2001) Invasion and persistent intracellular colonization of erythrocytes. A unique parasitic strategy of the emerging pathogen Bartonella. J Exp Med 193: 1077–1086. 11342592 10.1084/jem.193.9.1077 PMC2193435

[15] Engel P, Salzburger W, Liesch M, Chang CC, Maruyama S, et al (2011) Parallel Evolution of a Type IV Secretion System in Radiating Lineages of the Host-Restricted Bacterial Pathogen Bartonella. PLoS Genet 7: e1001296. 21347280 10.1371/journal.pgen.1001296 PMC3037411

[16] Seubert A, Hiestand R, de la Cruz F, Dehio C (2003) A bacterial conjugation machinery recruited for pathogenesis. Mol Microbiol 49: 1253–1266. 12940985 10.1046/j.1365-2958.2003.03650.x

[17] Schulein R, Dehio C (2002) The VirB/VirD4 type IV secretion system of Bartonella is essential for establishing intraerythrocytic infection. Mol Microbiol 46: 1053–1067. 12421311 10.1046/j.1365-2958.2002.03208.x

[18] Dehio C (2008) Infection-associated type IV secretion systems of Bartonella and their diverse roles in host cell interaction. Cell Microbiol 10: 1591–1598. 18489724 10.1111/j.1462-5822.2008.01171.x PMC2610397

[19] Vayssier-Taussat M, Le Rhun D, Deng HK, Biville F, Cescau S, et al (2010) The Trw type IV secretion system of Bartonella mediates host-specific adhesion to erythrocytes. PLoS Pathog 6: e1000946. 20548954 10.1371/journal.ppat.1000946 PMC2883598

[20] Nystedt B, Frank AC, Thollesson M, Andersson SG (2008) Diversifying selection and concerted evolution of a type IV secretion system in Bartonella. Mol Biol Evol 25: 287–300. 18065487 10.1093/molbev/msm252

[21] Schulein R, Guye P, Rhomberg TA, Schmid MC, Schroder G, et al (2005) A bipartite signal mediates the transfer of type IV secretion substrates of Bartonella henselae into human cells. Proc Natl Acad Sci U S A 102: 856–861. 15642951 10.1073/pnas.0406796102 PMC545523

[22] Selbach M, Paul FE, Brandt S, Guye P, Daumke O, et al (2009) Host cell interactome of tyrosine-phosphorylated bacterial proteins. Cell Host Microbe 5: 397–403. 19380118 10.1016/j.chom.2009.03.004

[23] Schmid MC, Schulein R, Dehio M, Denecker G, Carena I, et al (2004) The VirB type IV secretion system of Bartonella henselae mediates invasion, proinflammatory activation and antiapoptotic protection of endothelial cells. Mol Microbiol 52: 81–92. 15049812 10.1111/j.1365-2958.2003.03964.x

[24] Rhomberg TA, Truttmann MC, Guye P, Ellner Y, Dehio C (2009) A translocated protein of Bartonella henselae interferes with endocytic uptake of individual bacteria and triggers uptake of large bacterial aggregates via the invasome. Cell Microbiol 11: 927–945. 19302579 10.1111/j.1462-5822.2009.01302.x

[25] Schmid MC, Scheidegger F, Dehio M, Balmelle-Devaux N, Schulein R, et al (2006) A translocated bacterial protein protects vascular endothelial cells from apoptosis. PLoS Pathog 2: e115. 17121462 10.1371/journal.ppat.0020115 PMC1657063

[26] Dehio C, Meyer M, Berger J, Schwarz H, Lanz C (1997) Interaction of Bartonella henselae with endothelial cells results in bacterial aggregation on the cell surface and the subsequent engulfment and internalisation of the bacterial aggregate by a unique structure, the invasome. J Cell Sci 110 (Pt 18) 2141–2154. 9378764 10.1242/jcs.110.18.2141

[27] Truttmann MC, Rhomberg TA, Dehio C (2011) Combined action of the type IV secretion effector proteins BepC and BepF promotes invasome formation of Bartonella henselae on endothelial and epithelial cells. Cell Microbiol 13: 284–299. 20964799 10.1111/j.1462-5822.2010.01535.x

[28] Iwanicki MP, Vomastek T, Tilghman RW, Martin KH, Banerjee J, et al (2008) FAK, PDZ-RhoGEF and ROCKII cooperate to regulate adhesion movement and trailing-edge retraction in fibroblasts. J Cell Sci 121: 895–905. 18303050 10.1242/jcs.020941

[29] Rid R, Schiefermeier N, Grigoriev I, Small JV, Kaverina I (2005) The last but not the least: the origin and significance of trailing adhesions in fibroblastic cells. Cell Motil Cytoskeleton 61: 161–171. 15909298 10.1002/cm.20076

[30] Worthylake RA, Lemoine S, Watson JM, Burridge K (2001) RhoA is required for monocyte tail retraction during transendothelial migration. J Cell Biol 154: 147–160. 11448997 10.1083/jcb.200103048 PMC2196864

[31] Heasman SJ, Carlin LM, Cox S, Ng T, Ridley AJ (2010) Coordinated RhoA signaling at the leading edge and uropod is required for T cell transendothelial migration. J Cell Biol 190: 553–563. 20733052 10.1083/jcb.201002067 PMC2928012

[32] Fan H, Hall P, Santos LL, Gregory JL, Fingerle-Rowson G, et al (2011) Macrophage migration inhibitory factor and CD74 regulate macrophage chemotactic responses via MAPK and Rho GTPase. J Immunol 186: 4915–4924. 21411731 10.4049/jimmunol.1003713 PMC3388798

[33] Kim MJ, Kim S, Kim Y, Jin EJ, Sonn JK (2012) Inhibition of RhoA but not ROCK induces chondrogenesis of chick limb mesenchymal cells. Biochem Biophys Res Commun 418: 500–505. 22281493 10.1016/j.bbrc.2012.01.053

[34] Aktories K, Wilde C, Vogelsgesang M (2004) Rho-modifying C3-like ADP-ribosyltransferases. Rev Physiol Biochem Pharmacol 152: 1–22. 15372308 10.1007/s10254-004-0034-4

[35] Groeger G, Nobes CD (2007) Co-operative Cdc42 and Rho signalling mediates ephrinB-triggered endothelial cell retraction. Biochem J 404: 23–29. 17300218 10.1042/BJ20070146 PMC1868826

[36] Marignac G, Barrat F, Chomel B, Vayssier-Taussat M, Gandoin C, et al (2010) Murine model for Bartonella birtlesii infection: New aspects. Comp Immunol Microbiol Infect Dis 33: 95–107. 20097421 10.1016/j.cimid.2008.07.011

[37] Savina A, Amigorena S (2007) Phagocytosis and antigen presentation in dendritic cells. Immunol Rev 219: 143–156. 17850487 10.1111/j.1600-065X.2007.00552.x

[38] Shortman K, Naik SH (2007) Steady-state and inflammatory dendritic-cell development. Nat Rev Immunol 7: 19–30. 17170756 10.1038/nri1996

[39] Skinner JA, Pilione MR, Shen H, Harvill ET, Yuk MH (2005) Bordetella type III secretion modulates dendritic cell migration resulting in immunosuppression and bacterial persistence. J Immunol 175: 4647–4652. 16177111 10.4049/jimmunol.175.7.4647

[40] Zlokarnik G, Negulescu PA, Knapp TE, Mere L, Burres N, et al (1998) Quantitation of transcription and clonal selection of single living cells with beta-lactamase as reporter. Science 279: 84–88. 9417030 10.1126/science.279.5347.84

[41] Shields JD, Fleury ME, Yong C, Tomei AA, Randolph GJ, et al (2007) Autologous chemotaxis as a mechanism of tumor cell homing to lymphatics via interstitial flow and autocrine CCR7 signaling. Cancer Cell 11: 526–538. 17560334 10.1016/j.ccr.2007.04.020

[42] Edelman GM, Gally JA (2001) Degeneracy and complexity in biological systems. Proc Natl Acad Sci U S A 98: 13763–13768. 11698650 10.1073/pnas.231499798 PMC61115

[43] Larsen M, Tremblay ML, Yamada KM (2003) Phosphatases in cell-matrix adhesion and migration. Nat Rev Mol Cell Biol 4: 700–711. 14506473 10.1038/nrm1199

[44] Lamalice L, Le Boeuf F, Huot J (2007) Endothelial cell migration during angiogenesis. Circ Res 100: 782–794. 17395884 10.1161/01.RES.0000259593.07661.1e

[45] Insall RH, Machesky LM (2009) Actin dynamics at the leading edge: from simple machinery to complex networks. Dev Cell 17: 310–322. 19758556 10.1016/j.devcel.2009.08.012

[46] Ponti A, Machacek M, Gupton SL, Waterman-Storer CM, Danuser G (2004) Two distinct actin networks drive the protrusion of migrating cells. Science 305: 1782–1786. 15375270 10.1126/science.1100533

[47] Zaidel-Bar R, Ballestrem C, Kam Z, Geiger B (2003) Early molecular events in the assembly of matrix adhesions at the leading edge of migrating cells. J Cell Sci 116: 4605–4613. 14576354 10.1242/jcs.00792

[48] Wood W, Martin P (2002) Structures in focus–filopodia. Int J Biochem Cell Biol 34: 726–730. 11950590 10.1016/s1357-2725(01)00172-8

[49] Miao L, Vanderlinde O, Stewart M, Roberts TM (2003) Retraction in amoeboid cell motility powered by cytoskeletal dynamics. Science 302: 1405–1407. 14631043 10.1126/science.1089129

[50] Jermy A (2011) Bacterial pathogenesis: legionella effector under friendly fire. Nat Rev Microbiol 9: 80. 10.1038/nrmicro2509 21287709

[51] Chaturvedi LS, Marsh HM, Basson MD (2011) Role of RhoA and its effectors ROCK and mDia1 in the modulation of deformation-induced FAK, ERK, p38, and MLC motogenic signals in human Caco-2 intestinal epithelial cells. Am J Physiol Cell Physiol 301: C1224–1238. 21849669 10.1152/ajpcell.00518.2010 PMC3213924

[52] Narumiya S, Tanji M, Ishizaki T (2009) Rho signaling, ROCK and mDia1, in transformation, metastasis and invasion. Cancer Metastasis Rev 28: 65–76. 19160018 10.1007/s10555-008-9170-7

[53] Kubori T, Galan JE (2003) Temporal regulation of salmonella virulence effector function by proteasome-dependent protein degradation. Cell 115: 333–342. 14636560 10.1016/s0092-8674(03)00849-3

[54] Ingmundson A, Delprato A, Lambright DG, Roy CR (2007) Legionella pneumophila proteins that regulate Rab1 membrane cycling. Nature 450: 365–369. 17952054 10.1038/nature06336

[55] Saenz HL, Engel P, Stoeckli MC, Lanz C, Raddatz G, et al (2007) Genomic analysis of Bartonella identifies type IV secretion systems as host adaptability factors. Nat Genet 39: 1469–1476. 18037886 10.1038/ng.2007.38

[56] Koesling J, Aebischer T, Falch C, Schulein R, Dehio C (2001) Cutting edge: antibody-mediated cessation of hemotropic infection by the intraerythrocytic mouse pathogen Bartonella grahamii. J Immunol 167: 11–14. 11418625 10.4049/jimmunol.167.1.11

[57] Lopez-Bravo M, Ardavin C (2008) In vivo induction of immune responses to pathogens by conventional dendritic cells. Immunity 29: 343–351. 18799142 10.1016/j.immuni.2008.08.008

[58] Randolph GJ, Ochando J, Partida-Sanchez S (2008) Migration of dendritic cell subsets and their precursors. Annu Rev Immunol 26: 293–316. 18045026 10.1146/annurev.immunol.26.021607.090254

[59] Alvarez D, Vollmann EH, von Andrian UH (2008) Mechanisms and consequences of dendritic cell migration. Immunity 29: 325–342. 18799141 10.1016/j.immuni.2008.08.006 PMC2818978

[60] Gorvel JP (2008) Brucella: a Mr “Hide” converted into Dr Jekyll. Microbes Infect 10: 1010–1013. 18664389 10.1016/j.micinf.2008.07.007

[61] Salcedo SP, Marchesini MI, Lelouard H, Fugier E, Jolly G, et al (2008) Brucella control of dendritic cell maturation is dependent on the TIR-containing protein Btp1. PLoS Pathog 4: e21. 18266466 10.1371/journal.ppat.0040021 PMC2233671

[62] Dehio M, Knorre A, Lanz C, Dehio C (1998) Construction of versatile high-level expression vectors for Bartonella henselae and the use of green fluorescent protein as a new expression marker. Gene 215: 223–229. 9714815 10.1016/s0378-1119(98)00319-9

[63] Brasel K, De Smedt T, Smith JL, Maliszewski CR (2000) Generation of murine dendritic cells from flt3-ligand-supplemented bone marrow cultures. Blood 96: 3029–3039. 11049981

[64] Vigl B, Aebischer D, Nitschke M, Iolyeva M, Rothlin T, et al (2011) Tissue inflammation modulates gene expression of lymphatic endothelial cells and dendritic cell migration in a stimulus-dependent manner. Blood 118: 205–215. 21596851 10.1182/blood-2010-12-326447

