# Supplementary figures and images for: A Translocated Effector Required for Bartonella Dissemination from Derma to Blood Safeguards Migratory Host Cells from Damage by Co-translocated Effectors
Source: PLoS Pathog. 2014 Jun 19;10(6):e1004187. doi: 10.1371/journal.ppat.1004187 (PMC4063953; doi:10.1371/journal.ppat.1004187)

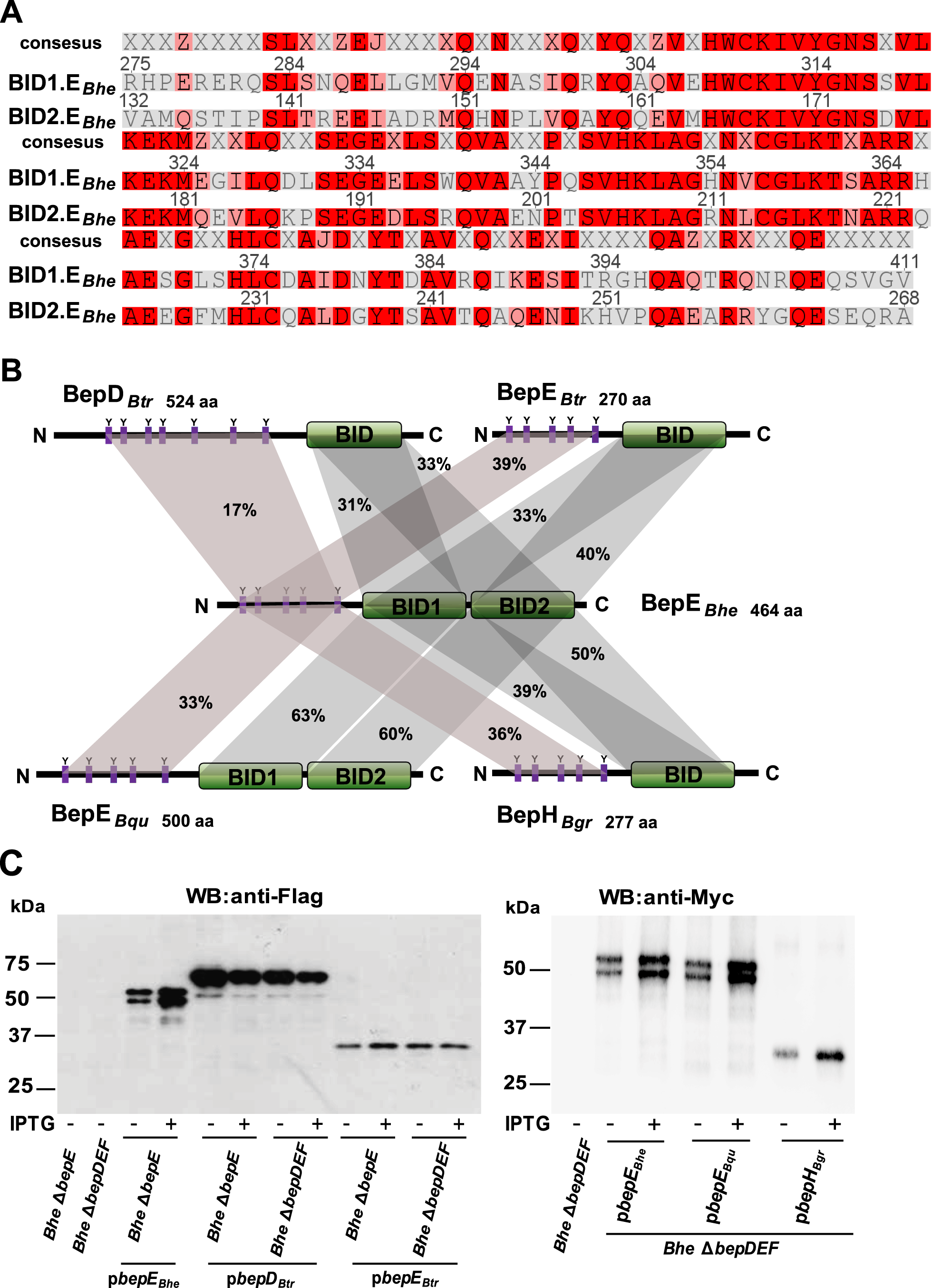

Supplement: Figure S1 — (A) Sequence alignment of BID1 (BID1.E Bhe ) and BID2 (BID2.E Bhe ) domains of BepE Bhe . The BepE BID domains were aligned using Geneious Pro 5.3.4. Identical aa are highlighted in red, similar aa in pink, and non-conserved aa in grey. (B) Domain organization of BepE orthologues in Btr, Bhe, Bqu and Bgr. The BepE homologues from Bartonella species depicted in the figure (BepEBhe, BepDBtr, BepEBtr, BepEBqu and BepHBgr) were aligned using Geneious Pro 5.3.4. The amino acid sequence alignment with pairwise % identity is indicated. The tyrosine-containing N-termini and BID domains were aligned independently. (C) Protein levels of the BepEBhe homologues, BepEBqu, BepEBtr, BepDBtr and BepHBgr by overexpression in Bhe ΔbepE and Bhe ΔbepDEF. The anti-Flag and anti-Myc western blots were obtained from total lysate of corresponding Bhe strains. (TIF) [file ppat.1004187.s001.tif]

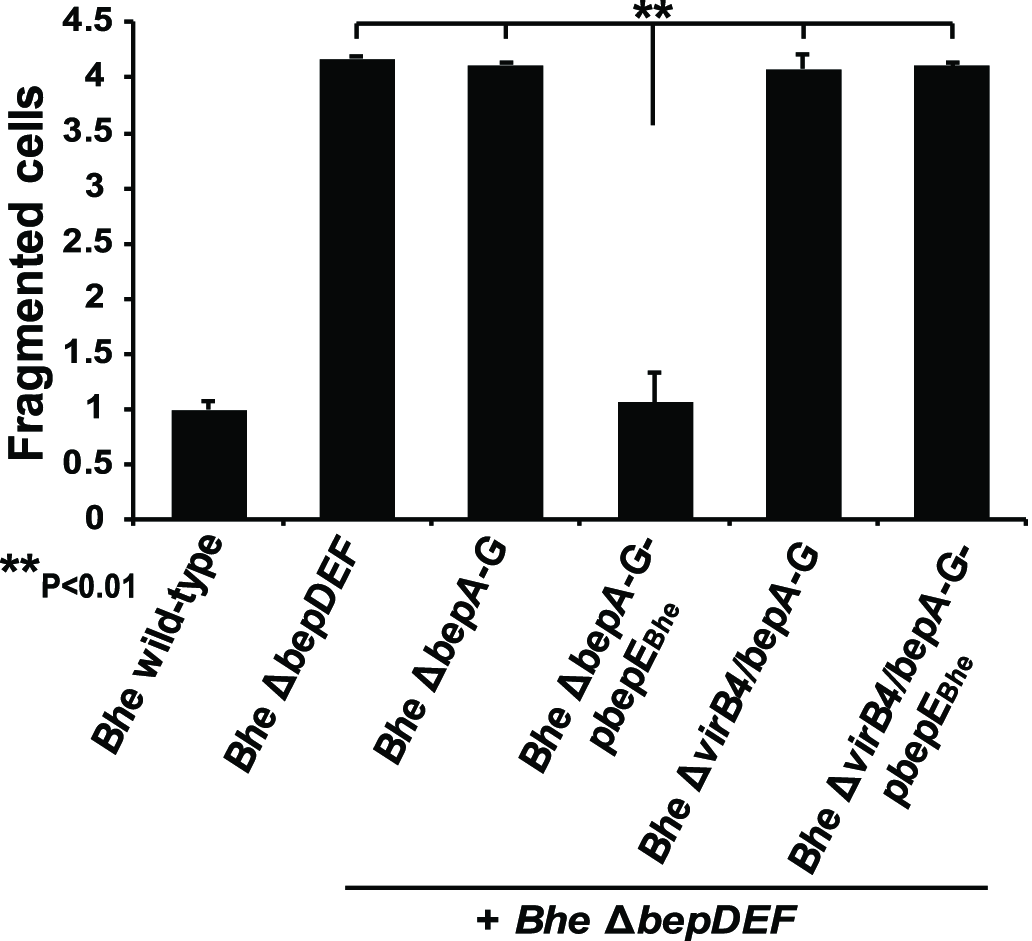

Supplement: Figure S2 — BepE inhibits host cell fragmentation upon translocation via T4SS. Subconfluent monolayers of HUVECs were infected with MOI = 200 or MOI = 200+200 in case of mixed infection depicted in the figure. Quantification of cell fragmentation at 48 h post infection was performed as described for Fig. 1C and D and presented as mean of triplicate samples +/− SD. Statistical significance was determined using Student's t-test. P<0.05 was considered statistically significant. Data from one representative experiment (n = 2) are presented. (TIF) [file ppat.1004187.s002.tif]

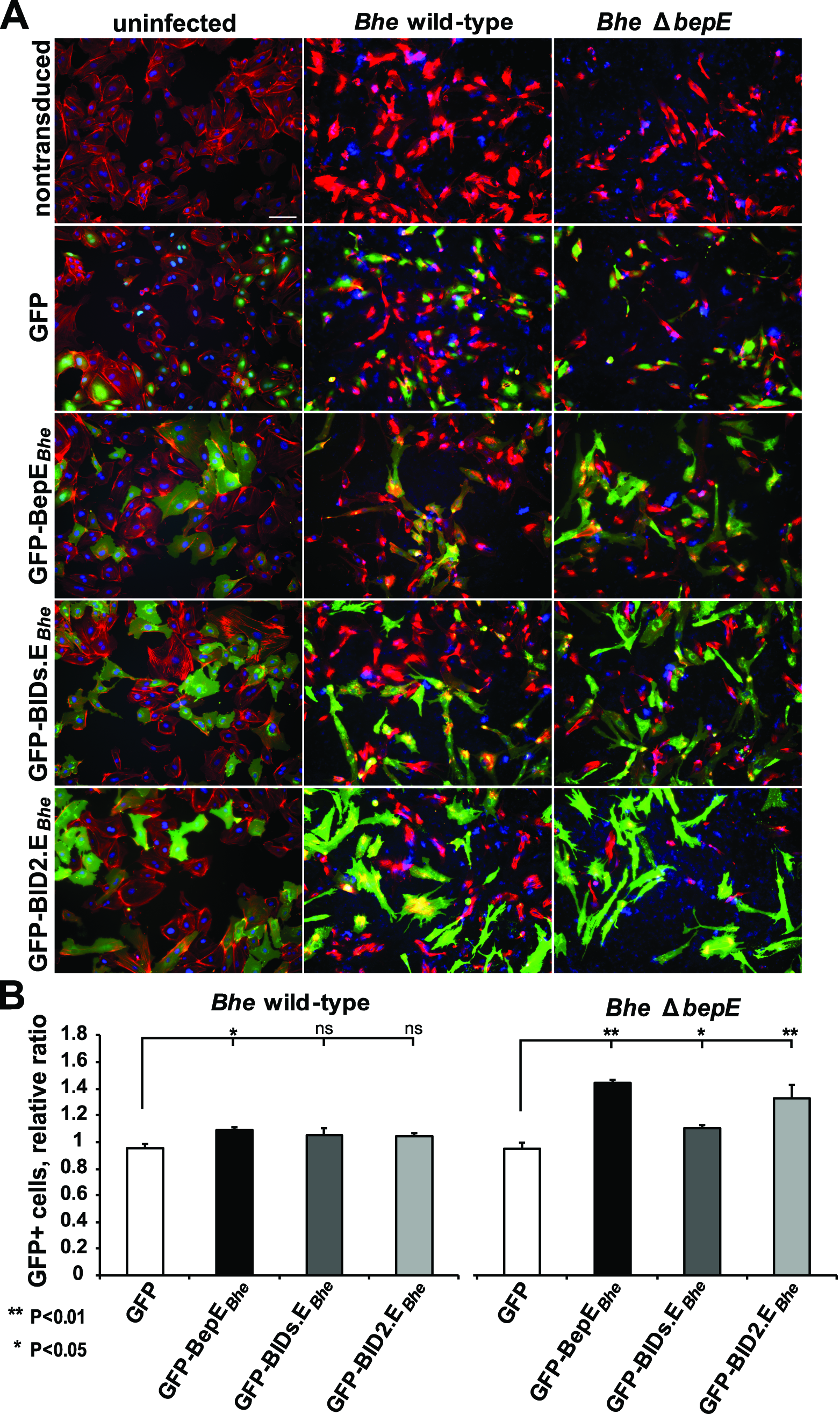

Supplement: Figure S3 — Ectopic expression of BepE Bhe protects HUVECs from cell fragmentation. (A, B) HUVECs of an early passage were transduced with lentiviruses for the expression of the depicted GFP-fusion proteins. The mixed culture of transduced and non-transduced cells were infected with the indicated Bhe strains (MOI = 200). Infected cells were either fixed and stained for microscopy or analyzed for the survival by FACS at 48 hpi. (A) Representative microscopy images (scale bar = 100 µm). F-actin is represented in red (Phalloidin), DNA in blue (DAPI), GFP in green. (B) Protection by GFP-fused BepE and its derivatives against fragmentation induced by Bhe strains. GFP-positive cell were quantified by FACS and normalized to the uninfected cell population. One representative experiment (n = 3) with the mean of triplicate samples +/− SD are presented. Statistical significance was determined using Student's t-test. P<0.05 was considered statistically significant. (TIF) [file ppat.1004187.s003.tif]

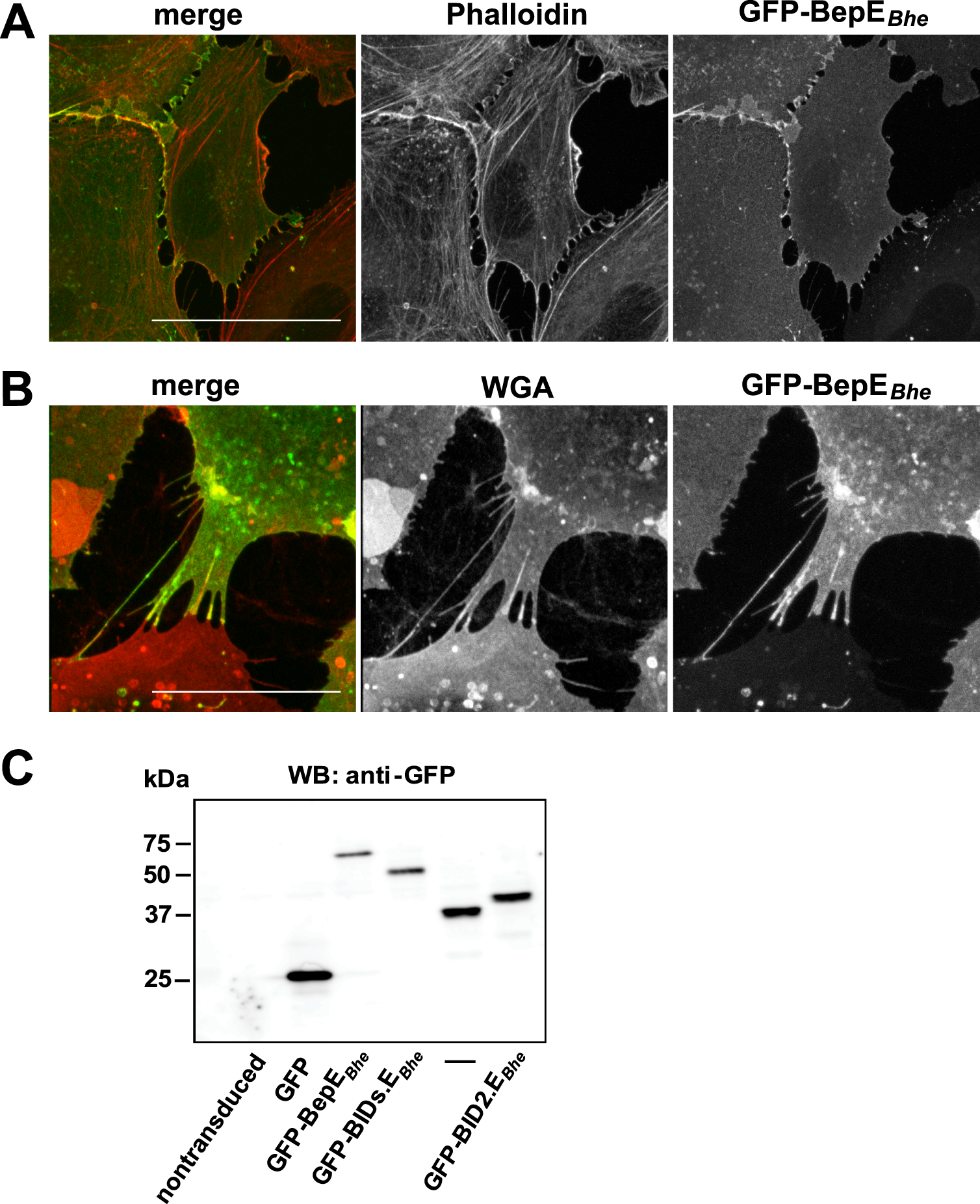

Supplement: Figure S4 — Ectopically expressed GFP-BepE Bhe localizes to cell-to-cell contacts. (A) (B) HUVECs of an early passage were transduced with lentiviruses directing expression of GFP-BepEBhe. Cells were fixed and stained by Phalloidin (Scale bar = 50 µm) (A) or stained by wheat germ agglutinin (WGA) and fixed afterwards (Scale bar = 25 µm) (B). Samples were subjected to confocal microscopy. (C) Lentivirally transduced HUVECs were tested for the expression of respective GFP-fusion proteins. Total-cell extracts were separated by SDS-PAGE and blotted with anti-GFP antibodies. (TIF) [file ppat.1004187.s004.tif]

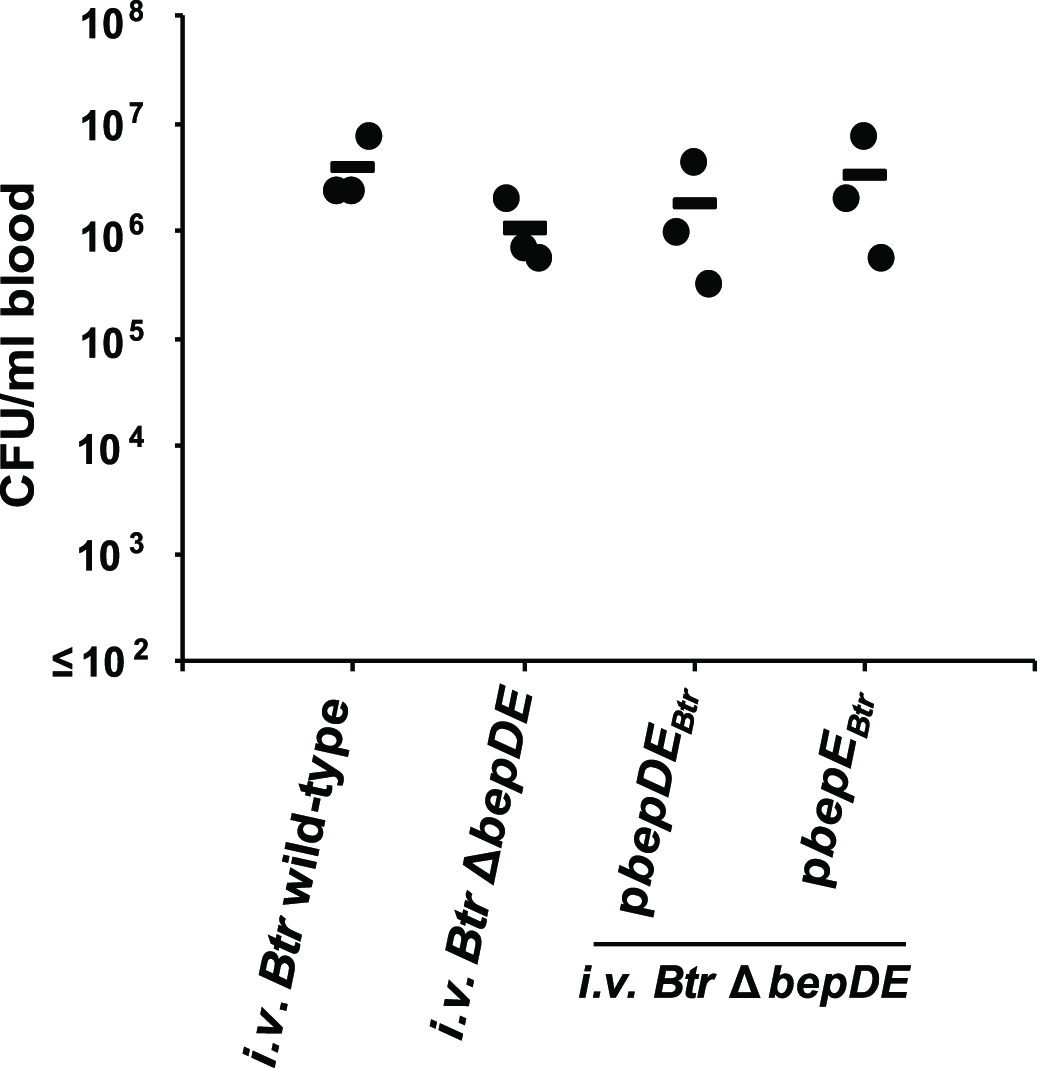

Supplement: Figure S5 — Rat intravenous ( i.v ) infection by Btr Δ bepDE . Groups of rats (n = 3) were injected in the tail vein (i.v.) with the depicted Btr strains. Blood was drawn at seven dpi, diluted and plated on sheep blood supplemented Columbia agar plates (CBA) for counting colony forming units (CFU). The graph represents CFUs/ml of blood for individual animals (circles) and their cohort mean (line). (TIF) [file ppat.1004187.s005.tif]

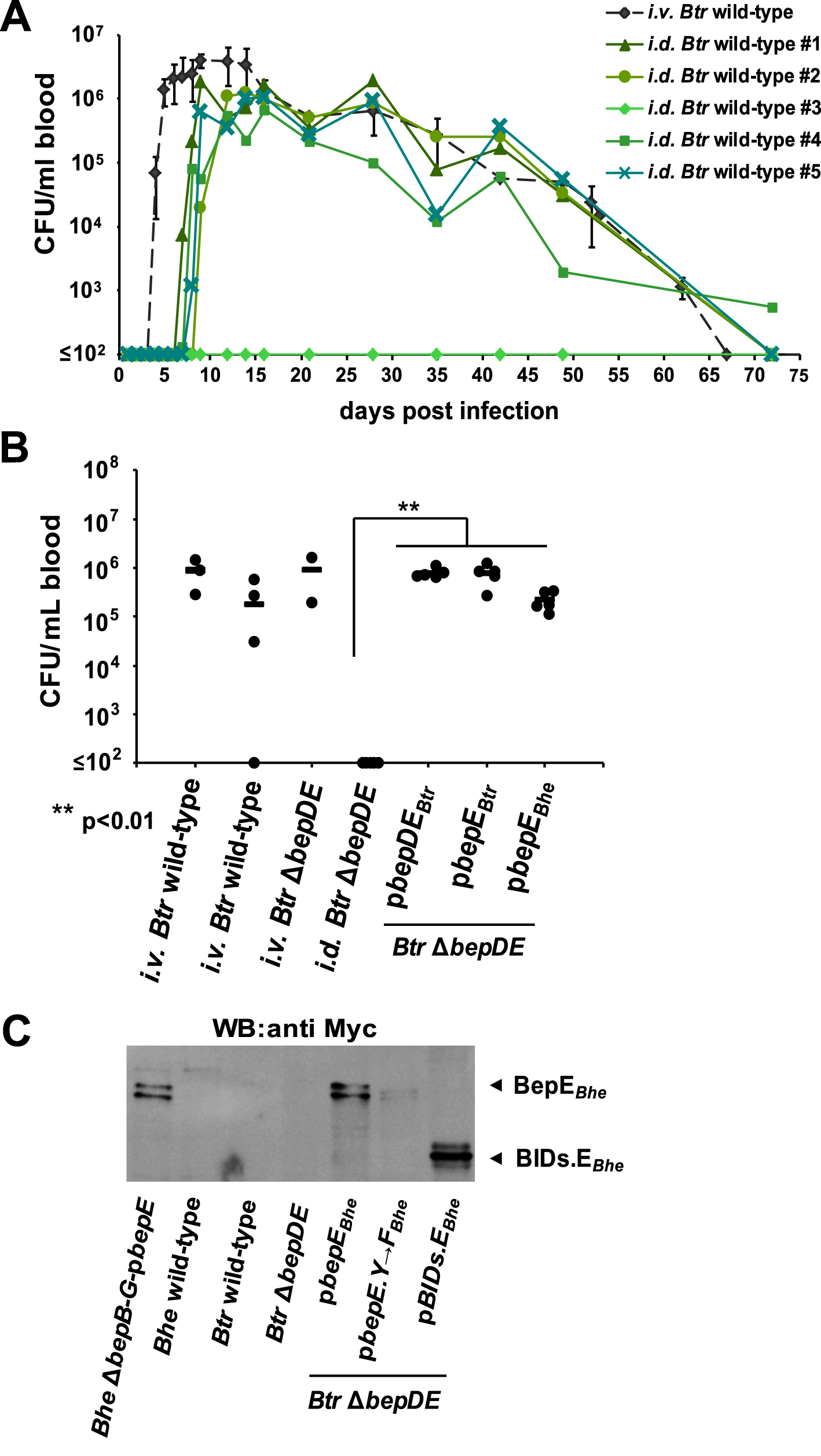

Supplement: Figure S6 — (A) Comparison of rat blood colonization by Btr wild-type after intradermal ( i.d. ) and intravenous ( i.v. ) infections. Groups of rats (n = 5) were injected in the tail vein (i.v.) or in the ear dermis (i.d.) with Btr wild-type. Blood was drawn, diluted and plated on sheep blood supplemented Columbia agar plates (CBA) for counting of colony forming units. Bacteremia (per ml of blood) of Btr wild-type i.d.-infected single animals is compared to the i.v. infection (dashed line, mean of 6 animals) described previously by Schulein et al, 2001. (B) Complementation of the Btr ΔbepDE mutant with BepE is sufficient to restore bacteremia in rats infected by the i.d. route. Groups of rats (n≥3) were infected with the indicated strains by the i.v. or i.d. route. Blood was drawn at 10 dpi and CFUs were recovered as described for (A). The graph represents CFUs/ml of blood for individual animals (circles) and their cohort average (line). Statistical significance was determined using Student's t-test. P<0.05 was considered statistically significant. (C) Protein levels of the BepEBtr homologue, BepEBhe and its mutants by overexpression in Btr ΔbepDE. The anti-Myc western blots were obtained from total lysate of corresponding Bartonella strains. (TIF) [file ppat.1004187.s006.tif]
