# Supplementary material for: A Translocated Effector Required for Bartonella Dissemination from Derma to Blood Safeguards Migratory Host Cells from Damage by Co-translocated Effectors
Source: PLoS Pathog. 2014 Jun 19;10(6):e1004187. doi: 10.1371/journal.ppat.1004187 (PMC4063953; doi:10.1371/journal.ppat.1004187)
Supplement: Table S1 — Bacterial strains and plasmids used in this study. (DOCX) [file ppat.1004187.s007.docx]

**Table S1: Bacterial strains and plasmids used in this study**

**Strain or plasmid Genotype or relevant characteristics Reference**

**Plasmids**

pRS40 vector for expression of [[1](#_ENREF_1)]

NLS-Cre-MobA fusion proteins

pRS25 vector to generate in-frame mutant deletion of *virB4* [[2](#_ENREF_2)]

pCD353 Bartonella spp. vector, encoding GFP [[3](#_ENREF_3)]

pBR322 broad host range resistance plasmid Bolivar, *et al*.1997

pPG100 *E.coli*-*Bartonella* shuttle vector, [[1](#_ENREF_1)]

encoding a short Flag tag

pTR1000 Basic mutagenesis vector to generate in-frame [[1](#_ENREF_1)]

mutants in RSE247 and RSE149

pPG110 *E.coli*-*Bartonella* shuttle vector, This work

for tag-less expression

pPG180 *E.coli*-*Bartonella* shuttle vector, This work

encoding a short Myc tag

pPG162 derivative of pTR1000 to generate in-frame [[4](#_ENREF_4)]

mutants of RSE247

pPG163 derivative of pTR1000 to generate in-frame This work

mutants of RSE247

pPG611 derivative of pTR1000 to generate pPG612 [[5](#_ENREF_5)]

pPG612 derivative of pTR1000 to generate chromosomal This work

insertion to RSE247

pFS20 derivative of pTR1000 to generate in-frame This work

mutants of RSE149

pPG104 encoding Flag-BepD*_Bhe_* [[1](#_ENREF_1)]

pPG105 encoding Flag -BepE*_Bhe_* [[6](#_ENREF_6)]

pPG106 encoding Flag -BepF*_Bhe_* [[6](#_ENREF_6)]

pPG185 encoding Myc-BepE*_Bhe_* This work

pPG185.M118 encoding Myc-BepE.Y­🡪F*_Bhe_* This work

pPG172 encoding Myc-BIDs.E*_Bhe_* This work

pRO104 encoding Myc-BID2.E*_Bhe_* This work

pRO105 encoding Myc-BepE*_Bqu_* This work

pRO107 encoding Myc-BepH*_Bgr_* This work

pFS32 encoding BepE*_Btr_* This work

pFS33 encoding BepD*_Btr,,_*BepE*_Btr_* This work

pFS01 encoding Flag -BepD*_Btr_* This work

pFS02 encoding Flag -BepE*_Btr_* This work

**Eukaryotic expression plasmids**

pCMV-Flag2 eukaryotic expression vector, Eastman Kodak, encoding a short Flag tag New Haven

pWay21 plasmid for ectopic expression of Molecular Motion

N-terminal eGFP fusion proteins Montana lab

pAP014 HA-eGFP- BepB*_Bhe_* [[7](#_ENREF_7)]

pPG1173.BIDs.E*_Bhe_* encoding Flag-BIDs.E*_Bhe_* This work

pRO1000 encoding eGFP-BepE*_Bhe_* This work

pRO1106 encoding Flag-eGFP-BIDs.E*_Bhe_* This work

pRO1107 encoding Flag-BID2.E*_Bhe_* This work

pRO1108 encoding Flag-eGFP-BID2.E*_Bhe_* This work

**Lenti viral plasmids**

pRRL-SV40(puro)_CMV(mcs) M. Kaeser

pLifeact-mCherry encoding LifeAct-mCherry O. Pertz, unpublished

pRO300 encoding eGFP This work

pRO301 encoding eGFP-BepE*_Bhe_* This work

pRO302 encoding eGFP-BIDs.E*_Bhe_* This work

pRO304 encoding eGFP-BID2.E*_Bhe_* This work

pMDL packaging vector for pRRL-SV40(puro)_CMV(mcs) O. Pertz

pREV packaging vector for pRRL-SV40(puro)_CMV(mcs) O. Pertz

pVSVG packaging vector for pRRL-SV40(puro)_CMV(mcs) O. Pertz

***E. coli* strains**

NovaBlue endA1 hsdR17(r K12–m K12+) supE44 thi-1 recA1 Novagen, Madison gyrA96 relA1 lac[F’ proA+B+ lacIqZΔM15::Tn10 (Tc^R^)]

β2150 F' lacZDM15 lacIq traD36 proA+B+ thrB1004 pro [[4](#_ENREF_4)]

thi strA hsdS lacZΔM15 ΔdapA::erm (Erm^R^) pir

***Bartonella henselae* strains**

RSE247 Spontaneous Sm^R^ strain of ATCC 49882T, [[1](#_ENREF_1)]

serving as *Bhe* wild-type

MTB012 RSE247 containing pCD353 [[8](#_ENREF_8)]

RSE242 *Bhe* Δ*virB4* mutant, derivative of RSE247 [[2](#_ENREF_2)]

MSE150 *Bhe* Δ*bepA-G* mutant, derivative of RSE247 [[1](#_ENREF_1)]

ROB311 *Bhe* Δ*bepA-G* mutant, containing pPG185 This work

LUB261 *Bhe* Δ*virB4/*Δ*bepA-G* mutant, This work

derivative of MSE150

LUB262 *Bhe* Δ*virB4/*Δ*bepA-G* mutant, containing pPG185 This work

PGC80 *Bhe* Δ*bepD* mutant, derivative of RSE247 [[4](#_ENREF_4)]

PGD20 *Bhe* Δ*bepE* mutant, derivative of RSE247 [[4](#_ENREF_4)]

TRB222 *Bhe* Δ*bepF* mutant, derivative of RSE247 [[4](#_ENREF_4)]

PGD29 *Bhe* Δ*bepDEF* mutant, derivative of RSE247 This work

TRB288 *Bhe* Δ*bepC* mutant, derivative of RSE247 [[4](#_ENREF_4)]

ROB322 *Bhe* Δ*bepCE* mutant, derivative of TRB288 This work

ROB320 *Bhe* Δ*bepE* mutant, containing pPG180 This work

PGE22 *Bhe* Δ*bepE* mutant, containing pPG185 This work

ROB109 *Bhe* Δ*bepE* mutant, containing pPG172 This work

ROB111 *Bhe* Δ*bepE* mutant containing pRO104 This work

PGD67 *Bhe* Δ*bepDEF* mutant, containing pPG185 This work

PGD43 *Bhe* Δ*bepDEF* mutant, containing pPG104 This work

PGD44 *Bhe* Δ*bepDEF* mutant, containing pPG105 This work

PGD45 *Bhe* Δ*bepDEF* mutant, containing pPG106 This work

ROB145 *Bhe* Δ*bepDEF* mutant containing pRO105 This work

ROB149 *Bhe* Δ*bepDEF* mutant containing pRO107 This work

ROB161 *Bhe* Δ*bepDEF* mutant containing pFS01 This work

ROB163 *Bhe* Δ*bepDEF* mutant containing pFS02 This work

PGH75 chromosomal integration of This work

*Bla-BIDBepD_Bhe_*, derivative of RSE247

***Bartonella tribocorum* strains**

RSE149 Spontaneous Sm^R^ strain, [[9](#_ENREF_9)]

serving as *Btr* wild-type

FS150 *Btr* Δ*bepDE* mutant, derivative of RSE149 This work

FS179 *Btr* Δ*bepDE* mutant, containing pFS32 This work

FS180 *Btr* Δ*bepDE* mutant, containing pFS33 This work

ROA150 *Btr* Δ*bepDE* mutant, containing pPG185 This work

ROA158 *Btr* Δ*bepDE* mutant, containing pPG185.M118 This work

ROA168 *Btr* Δ*bepDE* mutant, containing pPG172 This work

***Bartonella quintana strain***

RSE356 Spontaneous Sm^R^ strain, [[10](#_ENREF_10)]

serving as *Bqu* wild-type

***Bartonella grahamii strain***

CHDE142 No 376, isolated from Microtus sp. [[11](#_ENREF_11)]

serving as *Bgr* wild-type

**References:**

1. Schulein R, Guye P, Rhomberg TA, Schmid MC, Schroder G, et al. (2005) A bipartite signal mediates the transfer of type IV secretion substrates of Bartonella henselae into human cells. Proc Natl Acad Sci U S A 102: 856-861.

2. Schmid MC, Schulein R, Dehio M, Denecker G, Carena I, et al. (2004) The VirB type IV secretion system of Bartonella henselae mediates invasion, proinflammatory activation and antiapoptotic protection of endothelial cells. Mol Microbiol 52: 81-92.

3. Dehio C, Meyer M, Berger J, Schwarz H, Lanz C (1997) Interaction of Bartonella henselae with endothelial cells results in bacterial aggregation on the cell surface and the subsequent engulfment and internalisation of the bacterial aggregate by a unique structure, the invasome. J Cell Sci 110 ( Pt 18): 2141-2154.

4. Scheidegger F, Ellner Y, Guye P, Rhomberg TA, Weber H, et al. (2009) Distinct activities of Bartonella henselae type IV secretion effector proteins modulate capillary-like sprout formation. Cell Microbiol 11: 1088-1101.

5. Quebatte M, Dick MS, Kaever V, Schmidt A, Dehio C (2013) Dual input control: Activation of the Bartonella henselae VirB/D4 Type IV secretion system by the stringent sigma factor RpoH1 and the BatR/BatS two component system. Mol Microbiol.

6. Rhomberg TA, Truttmann MC, Guye P, Ellner Y, Dehio C (2009) A translocated protein of Bartonella henselae interferes with endocytic uptake of individual bacteria and triggers uptake of large bacterial aggregates via the invasome. Cell Microbiol 11: 927-945.

7. Pulliainen AT, Pieles K, Brand CS, Hauert B, Bohm A, et al. (2012) Bacterial effector binds host cell adenylyl cyclase to potentiate Galphas-dependent cAMP production. Proc Natl Acad Sci U S A 109: 9581-9586.

8. Truttmann MC, Rhomberg TA, Dehio C (2011) Combined action of the type IV secretion effector proteins BepC and BepF promotes invasome formation of Bartonella henselae on endothelial and epithelial cells. Cell Microbiol 13: 284-299.

9. Schulein R, Seubert A, Gille C, Lanz C, Hansmann Y, et al. (2001) Invasion and persistent intracellular colonization of erythrocytes. A unique parasitic strategy of the emerging pathogen Bartonella. J Exp Med 193: 1077-1086.

10. Schmid MC, Scheidegger F, Dehio M, Balmelle-Devaux N, Schulein R, et al. (2006) A translocated bacterial protein protects vascular endothelial cells from apoptosis. PLoS Pathog 2: e115.

11. Koesling J, Aebischer T, Falch C, Schulein R, Dehio C (2001) Cutting edge: antibody-mediated cessation of hemotropic infection by the intraerythrocytic mouse pathogen Bartonella grahamii. J Immunol 167: 11-14.
