# Supplementary material for: A Translocated Effector Required for Bartonella Dissemination from Derma to Blood Safeguards Migratory Host Cells from Damage by Co-translocated Effectors
Source: PLoS Pathog. 2014 Jun 19;10(6):e1004187. doi: 10.1371/journal.ppat.1004187 (PMC4063953; doi:10.1371/journal.ppat.1004187)
Supplement: Table S2 — Oligonucleotides used in this study. (DOCX) [file ppat.1004187.s008.docx]

**Table S2: Oligonucleotides used in this study**

**Name Sequence^a^ Restriction sites**

prTR016 CGGGATCCttagatggcgaaagctattgcc *Bam*HI

prTR041 CGGGATCCGCACACTCTGTGGAAAAACC *Bam*HI

prTR051 CAACCATCCCCTCCGACTCCCACAAATCAAAAAGGA

prTR055 CACAAAAAATGGTGCTGGC

prTR056 GCTCTAGAGGCGTTACACAGGGAAGAG *Xba*I

prFS09 CGGGATCCGCTTTCGTCGTTGGGCACA *Bam*HI

prFS10 ATCTGTTTTATGTTTATGCCACTTTAGCCTGTAAGA

GCCAGTG

prFS11 CACTGGCTCTTACAGGCTAAAGTGGCATAAACATAA

AACAGAT

prFS12 CGGGATCCTTTGAATTTCCTGCATTTTTTCTT *Bam*HI

prPG090 CGCGAGCTCTTTAAGAAGGAGATATACATATGGGG *Sac*I. *Nde*I

ATGCCTGGCAGTTTAT

prPG091 CGCGAGCTCTAAAAGAAATCAGAACGCAGAAGCG *Sac*I

prPG101 GGAATTCCATATGTTAGATGGCGAAAGCTATTGC *Nde*I

prPG141 CGCGCTTATTAATGGAACAAAAACTTATTTCTGAA *Ase*I, *Nde*I

prPG145 CGCGCTTATTAATCATATGTTACATACCAAAGGCCA *Nde*I

prPG148 GGAATTCCATATGGTCGACTACCACGCCGGATCCAG *Nde*I, *Sal*I

CACAATACCATCTCTAACAAGAGAG  *Bam*HI

prPG149 GGAATTCCATATGTTAGATGGCGAAAGCTATTGC *Nde*I

prPG190 GCTCCGCCATCGCCGCT

prPG191 GGATTTGAACGTTGCGAAGC

prPG208 ATAAGAATGCGGCCGCGAGCACAATACCATCTCTAAC *Not*I

AAGAGAG

prPG209 CGGGATCCGTTAGATGGCGAAAGCTATTGC *Bam*HI

prPG212 CGCGGATCCACACGTGAGATATCTATAATTCTCTCTCTG *Bam*HI

prPG213 AGCCGCGGCAAGATCTTCTTCAGAAATAAGTTTTTGTTC *Sac*II

CATGCATGCTTCCTTTCAAGC

prPG214 CTTATTTCTGAAGAAGATCTTGCCGCGGCTCACCCAGAAA *Sac*II

CGCTGGTG

prPG215 TGCGGCCGCCCAATGCTTAATCAGTGAGGCAC *Not*I

prPG216 GATTAAGCATTGGGCGGCCGCTGGAGTTGGCCC *Not*I

AGGAAGG

prPG217 ACGCGTCGACACATTTCATAACACTTCTTGGCGCAC *Sal*I

prPG280 TCCCCCCGGGATGAAAAGAAATCAACCACCCC *Xma*I

prPG281 ATAAGAATGCGGCCGCTTAGATGGCGAAAGCTATTGCC *Not*I

prRO030 GGAATTCCATATGCATCCTGAAAGGGAGAGAC *Nde*I

prRO031 CTTGCGGCCGCGATGCATCCTGAAAGGG *Not*I

prRO033 GGAATTCCATATGAAAAAAAACCAACCATCCTC *Nde*I

prRO034 GGAATTCCATATGTTAGCTGGCGAAAGCTATTGC *Nde*I

prRO035 GGAATTCCATATGAAAAAAGATCACCCTCACCC *Nde*I

prRO036 GGAATTCCATATGTTAGTTGGCAAAAGCCATTTC *Nde*I

prRO055 GATTGTCGACGAATTCTTAACGCGATTGTTGTA *Eco*RI

GCC TCT CGG

prRO068 GATTGTCGACGAATTCTTACTTGTACAGCTCGTCCATG *Eco*RI

CCGAGAGTG

prRO072 GACTCTAGAGGATCCGCCACCATGGTGAGCAAG *Bam*HI

GGCGAGGAGCTGTTCAC

prRO073 GATTGTCGACGAATTCTTAGATGGCGAAAGCTATTGCC *Eco*RI

TTTGCTCC

^a^Restriction endonuclease cleavage sites are underlined
