# Supplementary material for: A Translocated Effector Required for Bartonella Dissemination from Derma to Blood Safeguards Migratory Host Cells from Damage by Co-translocated Effectors
Source: PLoS Pathog. 2014 Jun 19;10(6):e1004187. doi: 10.1371/journal.ppat.1004187 (PMC4063953; doi:10.1371/journal.ppat.1004187)
Supplement: Materials and Methods S1 — Description of DNA manipulations. (DOCX) [file ppat.1004187.s009.docx]

**Materials and Methods S1**

**DNA manipulations**

Plasmids used in this study are listed in Table S1, primers are listed in Table S2.

*Plasmids for expression of Myc-Beps.* As a first step, for constructing vector pPG110 expressing native proteins, the oligonucleotide primers prPG090 and prPG091 were used to amplify a 0.4 kb fragment containing the *rrnB* terminator from pRS40 template DNA. Using flanking *Sac*I-sites the generated fragment was inserted into the corresponding site of pRS40, yielding pPG110. Next, a 1.67 kb fragment containing the complete *bepD* gene and an N-terminal Myc tag was amplified using oligonucleotide primers prPG141 and prPG145 from chromosomal DNA of RSE247 as template. Using flanking *Nde*I-sites the amplified fragment was ligated into the corresponding site of pPG110, yielding pPG184. The empty Myc tag expression vector was generated by cutting out *bepD_Bhe_* with *Nde*I from pPG184, and re-ligating the vector, yielding pPG180.

By excising full length *bepE_Bhe_* from pPG105 with *Nde*I and inserting it into the respective sites in pPG180, the Myc-BepE*_Bhe_* expressing vector, pPG185, was constructed.

The vector expressing the two C-terminal BIDs of BepE*_Bhe_* without the N-terminus, was constructed by PCR amplifying a fragment of 1 kb from pPG105 with the primers prPG148 and prPG149. After digesting the fragment with *Nde*I, it was inserted in the respective site of pPG180, yielding pPG172.

The vector expressing the last C-terminal BID2.E*_Bhe_* domain of BepE*_Bhe_*, was constructed by PCR amplifying a fragment of 576 bp from pPG105 with the primers prRO030 and prPG101. After digesting the PCR product with *Nde*I, it was inserted in the respective site of pPG180, yielding pRO104.

The vector expressing the BepE*_Bqu_* was constructed by PCR amplifying a fragment of 1502 bp from boiled colony of *Bqu* RSE356 with the primers prRO033 and prRO034. After digesting the PCR product with *Nde*I, it was inserted in the respective site of pPG180, yielding pRO105.

The vector expressing the BepH*_Bgr_* was constructed by PCR amplifying a fragment of 831 bp from boiled colony of *Bgr* CHDE142 with the primers prRO035 and prRO036. After digesting the PCR product with *Nde*I, it was inserted in the respective site of pPG180, yielding pRO107.

*Plasmids for lentiviral expression.* The lentiviral vector expressing GFP was constructed by PCR amplifying a fragment of 756 bp from pWay21 using prRO072 and prRO68. The PCR fragment was recombined to lentiviral vector pRRL-SV40(puro)_CMV(mcs) (a gift from M. Kaeser) by homologous recombination with the In-Fusion PCR cloning system (Clontech), yielding pRO300.

The vector expressing GFP-BepE*_Bhe_*, pRO1000, was constructed by PCR amplifying a fragment of 1401 bp from chromosomal DNA of RSE247 using the primers prPG280 and prGP281. The PCR product was digested with *Xma*I and *Not*I, and ligated into previously *Xma*I/ *Not*I-cut pWay21, yielding pRO1000. The lentiviral vector expressing GFP-BepE*_Bhe_* was constructed by PCR amplifying a fragment of 2126 bp from pRO1000 using prRO072 and prRO73. The PCR fragment was recombined to lentiviral vector pRRL-SV40(puro)_CMV(mcs) by homologous recombination with the In-Fusion PCR cloning system (Clontech), yielding pRO301.

*BIDs.E_Bhe_*, 1 kb fragment, was amplified by PCR from chromosomal DNA of RSE247 using prPG208 and prPG209. The PCR product was cut with *Not*I and *BamH*I, and ligated into the corresponding sites of the pCMV-FLAG2 plasmid, yielding pPG1173.BIDs.E*_Bhe_*. The vector expressing Flag-GFP-BIDs.E*_Bhe_* was constructed by digesting pPG1173.BIDs.E*_Bhe_* with *Not*I and inserting *gfp* with flanking *Not*I sites taken from pAP014, yielding pRO1106. pRO1106 was used as a template for PCR amplification of 1725 bp *gfp-BIDs.E_Bhe_* fragment by prRO072 and prRO73 primers for homologous recombination with lentiviral vector pRRL-SV40(puro)_CMV(mcs), yielding pRO302.

The vector expressing Flag-BID2.E*_Bhe_* was constructed by amplifying 570 bp fragment from chromosomal DNA of RSE247 using prRO31 and prTR016, digesting with *Not*I and *BamH*I, and ligated into the corresponding sites of the pPG1173.BIDs.E*_Bhe_* precut with *Not*I and *BamH*I, yielding pRO1107. Next, the vector expressing Flag-GFP-BID2.E*_Bhe_* was constructed by cutting pRO1107 with *Not*I and inserting *gfp* with flanking *Not*I sites taken from pAP014, yielding pRO1108. The lentiviral vector expressing GFP-BID2.E*_Bhe_* was constructed by PCR amplifying a fragment of 1314 bp from pRO1108 using prRO072 and prRO73. The PCR fragment was recombined to lentiviral vector pRRL-SV40(puro)_CMV(mcs) by homologous recombination with the In-Fusion PCR cloning system (Clontech), yielding pRO304.

**Construction of in-frame deletions.** In-frame deletion mutants of *Btr* RSE149 and *Bhe* RSE247 were generated by a two-step gene replacement procedure as described (Schulein & Dehio, 2002; Schmid *et al*., 2004) [[1](#_ENREF_1),[2](#_ENREF_2)]. The basic mutagenesis vector pTR1000 was described before (Schmid *et al.*, 2004) [[2](#_ENREF_2)]. All mutagenesis plasmids harbor a cassette with the flanking regions of the in-frame deletion in the gene(s) of interest. This cassette was generated from two PCR fragments amplified from chromosomal DNA of *Btr* RSE149 or *Bhe* RSE247 as template, either by megaprime PCR or by conventional cloning.

pFS20 used for generating a *Btr* Δ*bepDE* in-frame mutant was constructed as follows. Oligonucleotide primers prFS09 and prFS10 amplified fragment 1 (736 bp, containing 356 bp of 5` end of *bepC_Btr_*) and prFS11 and prFS12 amplified fragment 2 (665 bp, containing 345 bp of 3` end of *bepH_Btr_*). Both fragments were combined by megaprime PCR with oligonucleotide primers prFS09 and prFS12, resulting in a fragment of 1.64 kb carrying an in-frame deletion in *bepDE_Btr_*. By using flanking *BamH*I sites, the fragment was inserted into the corresponding site of pTR1000, yielding pFS20. The use of pFS20 for gene replacement in RSE149 resulted in the *Btr* Δ*bepDE* mutant FS150.

Construction of the in-frame mutant *Bhe* Δv*irB4* has been described previously (Schmid *et al.,* 2004) [[2](#_ENREF_2)].

pPG163 used for generating a *Bhe* Δ*bepDEF* mutant was constructed as follows. Oligonucleotide primers prTR041 and prTR051 amplified fragment 1 (853 bp, containing 321 bp of 5` end of *bepC_Bhe_)* and prTR055 and prTR056 amplified fragment 2 (665 bp, containing 345 bp of 3` end of *bepG_Bhe_*). Both fragments were combined by megaprime PCR with oligonucleotide primers prTR041 and prTR056, resulting in a fragment of 1.67 kb carrying an in-frame deletion in *bepDEF_Bhe_*. By using flanking *BamH*I sites, the fragment was inserted into the corresponding site of pTR1000, yielding pPG163. The use of pPG163 for gene replacement in RSE247 resulted in the *Bhe* Δ*bepDEF* mutant PGD29.

The use of pPG162 for gene replacement in TRB288 resulted in the *Bhe* Δ*bepCE* mutant ROB322.

The use of pRS25 for gene replacement in MS150 resulted in the *Bhe* Δ*virB4*/Δ*bepA-G* LUB261.

**Chromosomal integrations.** Suicide vector pPG612 for chromosomal integration for *Bhe* was constructed as follows. The oligonucleotide primers prPG212 and prPG213 amplified the intergenic region of *Bhe* *bepC* to *bepD* with tag encoding Myc epitope and flanking *BamH*I site as fragment 1. Oligonucleotide primers prPG214 and prPG215 amplified the *beta-lactamase* gene from pBR322 with flanking *SacI*I and *Not*I sites as fragment 2. Oligonucleotide primers prPG216 and prPG217 amplified the BID domain and terminator downstream of *bepD* from RSE247 chromosomal DNA with flanking *Not*I and *Sal*I sites as fragment 3. All tree fragments were combined by megaprime PCR with oligonucleotide primers prPG212 and prPG217 resulting in a fragment of 1.96 kb. This fragment was digested with *BamH*I and *Sal*I and ligated into previously *BamH*I/*Sal*I-cut pPG611, yielding pPG612. The use of pPG612 for gene replacement in RSE247 resulted in the *Bhe* PGH75.

**Tyrosine to phenylalanine exchange mutant in BepE*_Bhe._*** To exchange the putatively phosphorylated tyrosines to phenylalanines in the N-terminus of BepE, we applied megaprime PCR to re-amplify the sequence coding for BepE from pPG185. Briefly, using prPG190 and prPG191 binding on the plasmid pPG185 and two corresponding primers annealing to the site to be mutated, two fragments with a sequence overlap in the mutation site were amplified by PCR. In a second step, these two fragments were joined by megaprime PCR using prPG190 and prPG191, yielding a fragment of 2.1 kb. This fragment was then cut with *Nde*I and inserted into the previously *Nde*I-cut pPG180 vector. The Y37F; Y64F; Y91F; Y106F; Y129F (BepE*_Bhe_*.Y->F) mutant was generated after sequential mutation rounds using pPG185 as a template on a first round.

**References:**

1. Schulein R, Dehio C (2002) The VirB/VirD4 type IV secretion system of Bartonella is essential for establishing intraerythrocytic infection. Mol Microbiol 46: 1053-1067.

2. Schmid MC, Schulein R, Dehio M, Denecker G, Carena I, et al. (2004) The VirB type IV secretion system of Bartonella henselae mediates invasion, proinflammatory activation and antiapoptotic protection of endothelial cells. Mol Microbiol 52: 81-92.
